# Genomic analysis reveals variant association with high altitude adaptation in native chickens

**DOI:** 10.1038/s41598-019-45661-7

**Published:** 2019-06-25

**Authors:** Hamed Kharrati-Koopaee, Esmaeil Ebrahimie, Mohammad Dadpasand, Ali Niazi, Ali Esmailizadeh

**Affiliations:** 1 Institute of Biotechnology, School of Agriculture, Shiraz University, Shiraz, Iran; 2 The University of Adelaide, School of Animal and Veterinary Sciences, Adelaide, South Australia Australia; 3 School of Information Technology and Mathematical Science, Division of Information Technology, Engineering and the Environment, University of South Australia, South Australia Adelaide, Australia; 4 Genomics Research Platform, School of Life Sciences, La Trobe University, Melbourne Victoria, Australia; 5 Department of Animal science, School of Agriculture, Shiraz University, Shiraz, Iran; 6 State Key Laboratory of Genetic Resources and Evolution, and Yunnan Laboratory of Molecular Biology of Domestic Animals, Kunming Institute of Zoology, Chinese Academy of Sciences No. 32 Jiaochang Donglu, Kunming, Yunnan 650223 P.R. China; 7 Department of Animal science, Faculty of Agriculture, Shahid Bahonar University of Kerman, Kerman, Iran

**Keywords:** Bioinformatics, DNA sequencing, Next-generation sequencing, Comparative genomics

## Abstract

Native chickens are endangered genetic resources that are kept by farmers for different purposes. Native chickens distributed in a wide range of altitudes, have developed adaptive mechanisms to deal with hypoxia. For the first time, we report variants associated with high-altitude adaptation in Iranian native chickens by whole genome sequencing of lowland and highland chickens. We found that these adaptive variants are involved in DNA repair, organs development, immune response and histone binding. Amazingly, signature selection analysis demonstrated that differential variants are adaptive in response to hypoxia and are not due to other evolutionary pressures. Cellular component analysis of variants showed that mitochondrion is the most important organelle for hypoxia adaptation. A total of 50 variants was detected in mtDNA for highland and lowland chickens. High-altitude associated with variant discovery highlighted the importance of *COX3*, a gene involved in cell respiration, in hypoxia adaptation. The results of study suggest that MIR6644-2 is involved in hypoxia and high-altitude adaptations by regulation of embryo development. Finally, 3877 novel SNVs including the mtDNA ones, were submitted to EBI (PRJEB24944). Whole-genome sequencing and variant discovery of native chickens provided novel insights about adaptation mechanisms and highlights the importance of valuable genomic variants in chickens.

## Introduction

Chickens (*Gallus gallus domesticus*) are, domestically, considered as widespread birds. Chickens have been domesticated in India and South-East of Asia^1^. They are used for different purposes including decoration, religions, cock fighting, and food production^2^. Village chickens are also considered as important sources of food in Iran. Therefore, farmers keep them and consequently, their morphology, behavior, physiology and adaptation to local environment conditions have been remarkably changed^3^.

Iran is a large country that contains a special range of altitudes and climates. Therefore, there is a remarkable genetic diversity of indigenous chickens in Iran^4^. However, there is no previous report on Iranian native chickens at the genomic level. The next generation sequencing (NGS) has become a tool to describe genomic features^5^. Investigating native chickens at the genomic level can clarify the genomic basis of their differences and the specific traits of indigenous chickens can also be precisely explored^6^.

Usually, hypoxia occurs in high-attitude and pathological conditions. Hypoxia refers to the lack of oxygen^7^, and the adaptation to hypoxia is a complex process contains biological pathways and gene networks^8^. Thereby, understanding genetic factors that underlie adaptation to high-altitude conditions could lead to a new source of knowledge in order to understand the adaptation process^9^.

There are studies carried out in order to identify the genetic factors for high-altitude adaptation of Tibetan chickens, Yak, Tibetan pigs and Tibetan people^10^. Results of these studies show that hypoxia is involved in cerebral edema, tumorigenesis, myocardial ischemia and diabetes in human being, and some genes including Endothelin 1 (*EDN1*), Erythropoietin (*EPO*) and Aldosterone synthase (*CYP11B2*) are reported as the key genes of hypoxia adaptation^11–14^.

Recent evidences indicate that highland chickens are adapted based on factors including the small body size, the ability to outstand foraging, high hatchability, large organs (liver, heart and lungs), and higher hemoglobin concentration. Furthermore, the positive selection of highland populations is related to the cardiovascular and respiratory system development, responses to the radiation, inflammation, DNA repair, and the immune responses^15^. Recently, one of the most significant discussions about high-altitude condition is known as the mitochondrion organelle, because there is a close association between the lack of oxygen and cell respiration. In a previous study, nine single nucleotide polymorphisms (SNP) of *MT-COI* (mitochondria-cytochrome c oxidase subunit I) gene were detected for the high-altitude adaptation, but none of them was a missense mutation^16^.

In the study herein, the whole genome sequencing of highland chickens (Isfahan, Altitude = 2087 m) and lowland chickens (Mazandaran, Altitude = 54 m) was carried out in order to discover genomic variants, associated with high-altitude adaptation.

## Results

### Quality control

Results of the quality control indicate that short reads were sequenced based on an appropriate form. Trimming was carried out according to the Phred score and the nucleotide contribution. Removing at least ten primary bases, we trimmed 3′ side of short reads in order to minimize mapping errors. In addition, 5% of reads that contained the lowest Phred scores were also removed.

### Phenotypic data, phylogenetic and population structure analysis

Results of principle component analysis indicated that six components (combinations of traits) characterized 92 percent of the total variance; therefore, these components were selected in order to be utilized in the discriminate analysis (Table 1). Results of the discriminate analysis showed that collected lowland and highland samples can be classified into two separated groups, phenotypically. The accuracy was estimated to be 75% (Table 2).

**Table 1:** Principle component analysis of phenotypic traits for discriminate analysis and clustering the highland and lowland populations.

Component	1	2	3	4	5	6
Eigenvalue	6.45	2.64	2.07	1.14	0.79	0.69
Proportion	0.43	0.17	0.13	0.07	0.05	0.04
Cumulative	0.43	0.60	0.74	0.82	0.87	**0**.**92**

Twenty-four quantitative traits were measured on 16 highland and lowland chickens by our investigation. In order to reduce the measurement error, all chickens were mature while recording and the same recording protocol was also utilized in order to investigate the phenotypic traits of all the birds. Results of PCA show that six components of traits characterized 92 percent of total variance. These six components were selected for the discriminate analysis and classification of the studied population based on phenotypic traits.

**Table 2:** The classification of highland and lowland chickens based on the discriminate analysis.

Put into group	True group	
	Lowland	Highland
Lowland	8	2
Highland	2	4
Total sample	10	6
Correct sample	8	4
Proportion	0.80	0.66
Proportion correct	0.75	

Results of PCA (Table 1) were used for the discriminate analysis. Based on our findings, collected lowland and highland samples can be classified phenotypically into two separated groups. For instance, eight of ten lowland chickens are classified in the lowland chicken group, correctly. Similarly, two of six highland chickens are classified in the highland group. Also, the total accuracy of classification is estimated to be 75%.

Nei’s genetic distance between highland and lowland populations was estimated to be 3 percent. Thus, results of phylogenic analysis revealed that there was no significant genetic distance between highland and lowland populations.

**Figure 1:**
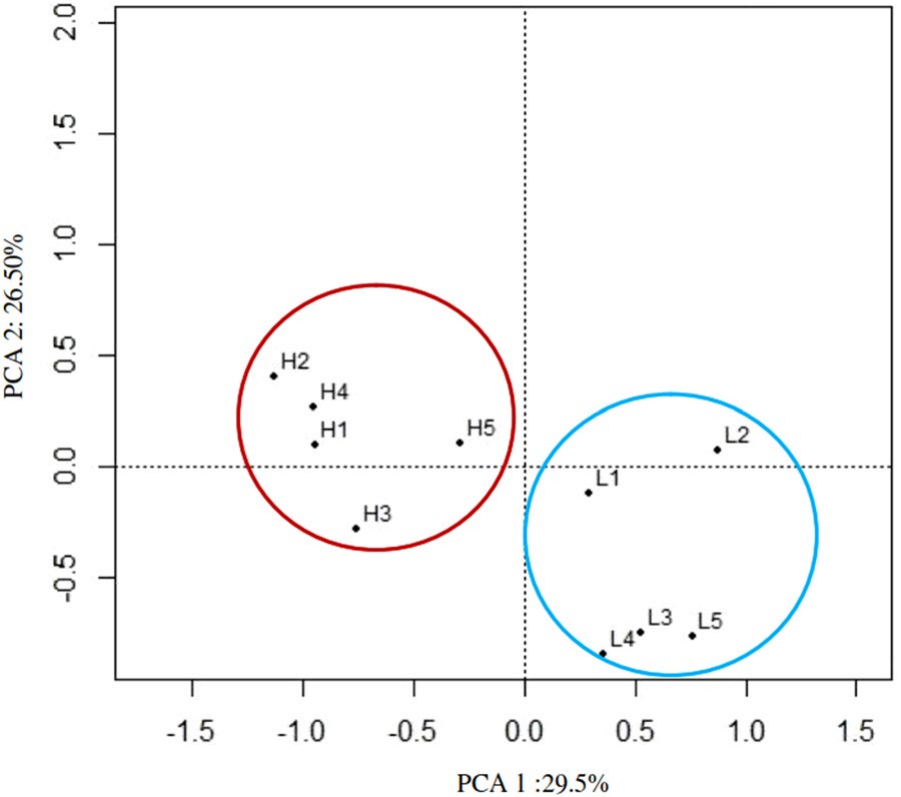
Classification of the highland and lowland chickens according to the results of multiple corresponding analyses (H: highland and L: lowland). The information of 10000 SNVs genotypes were utilized in order to classify the highland and lowland chickens based on multiple corresponding analysis by R program and Ca package. Results show that highland and lowland chickens can be divided into two groups based on their geographical distribution.

The analysis of genetic population structure was carried out by multiple correspondence analysis (MCA) based on 10000 SNVs genotypes. The first two components of variants were 29.5% and 26.5%, respectively. Results indicated that highland chickens were classified closely; while lowland chickens were classified together. Consequently, highland and lowland chickens could be classified into two groups (Fig. 1).

### Mapping, variants discovery and statistical analysis

The mapping efficiency versus reference genome was estimated between 94–97% for samples (Table 3). Parameters were optimized in order to decrease false variants for the variants discovery, (See Supplementary Table S1 for optimization process). The process of optimization was carried out in three steps, and parameters of the step 3 were selected in order to be utilized in the variants detection.

**Table 3:** The summary of short reads alignments against reference genome for highland and lowland native chickens.

Samples	Total reads	Mapped reads%	Reads in Pair%	Coverage before trimming	Coverage after trimming
Lowland	72462326	96.13	92.26	9.00	8.10
Lowland	70421388	97.00	93.53	8.70	7.88
Lowland	72850232	96.14	92.03	9.10	7.93
Lowland	62424846	96.01	92.08	7.70	6.82
Lowland	64804044	96.59	93.01	8.10	7.27
Highland	69105254	96.54	93.07	8.60	7.55
Highland	71837220	96.91	93.61	9.00	7.96
Highland	74894416	95.34	91.45	9.30	8.08
Highland	67495174	94.35	90.75	8.40	7.26
Highland	70314734	95.58	92.41	8.70	7.73

After trimming, the short reads of highland and lowland chickens were mapped against the standard reference genome of chicken (*Gallus gallus*, Ensembl-release 84). Mapping efficiency was estimated between 94–97% for samples, and the coverage analysis was carried out based on the lander and waterman equation.

We found that more than 20 million variations were generated, including single nucleotide variant (SNV), multi nucleotide variant (MNV), insertion, deletion and replacement (See Supplementary Table S2). Results of the statistical analysis indicated that there was a significant (*P* < 0.001) difference of variant distribution between highland and lowland samples (Table 4). Thus, we can realize that harsh environmental conditions, including the lack of oxygen, impacted on the variant distribution. In addition, the Chi-square test was carried out for each chromosome, separately. Most of chromosomes had significant differences in harboring associated variants with altitude alteration; therefore, those chromosomes that do not have significant differences are given in Table 5, including mtDNA, W, 4, 11, and 32.

**Table 4:** Fisher exact test for the identification of differences in variants distribution between highland and lowland samples.

Variants	P-value
SNV^***^	0.0012
MNV^***^	0.0001
Insertion^***^	0.0001
Deletion^***^	0.0001
Replacement^***^	0.0002

^***^(P < 0.001).

In order to analyze differences of variants distribution, the input file of variants was constructed based on the IDEG6 website instruction and statistical analysis was performed for each variant between highland and lowland samples, separately. Results indicated that variants distributions between highland and lowland were significant, and a possible explanation might also be defined as: differences in variants distribution can be affected by environmental conditions including high- altitude condition.

**Tablee 5:** Chi-square test to analyze the differential distributed variations between highland and lowland chickens for chromosomes (non-significant chromosomes are presented).

Variants and chromosomes						
Sex	Male	SNV	MNV	Insertion	Deletion	Replacement
		5,Mt	9, 11, 12, 18, 19, 25, 32	8, 10, 13, 19 21, 25, Mt	9, 10, 16, 18, 22, 25	6, 9, 10, 11, 12, 13, 14, 15, 16, 17, 18, 20, 22, 24, 26, 27, Mt
	Female	Mt	11, 32	Mt	—	3, 4, 11, W

Mt: Mitochondria DNA.

In addition to the statistical distribution analysis of each variant (Table 4), we analyzed the differential distributed variations between highland and lowland chickens for each chromosome incorporating the sex of birds in this study. We found that the distribution of variants was significant for the most of the chromosomes; therefore, to summarize, non-significant chromosomes were only presented in Table 5.

### Differential variants of highland and lowland ecotypes

In order to remove the common variants between highland and lowland chickens, total variants of those samples were compared. Differential variants were also kept in order to be utilized in further analyses (Table S3). Finally, the frequency thresholds of 50% and 100% were determined in order to carry out comparisons between the groups of males and females (Table S4). The threshold frequency is considered as the percentage of samples that contain variants. Generally, 97610 and 17024 variants were detected for both males and females as the differential variants of highland and lowland chickens, respectively. For the first time, 16623 and 3024 variants were identified as new variants in males and females. Results of filtering in the male birds, based on the overlap, showed that 614 and 96995 variants were located in coding and non-coding regions. We found that 202 out of 614 coding variants will lead to amino acid changes. It was observed that fewer coding and non-coding variants were detected among female birds (Table 6).

**Table 6:** Classification of differential variants in different types of categories.

Sex	Total variants	Novel variants	Coding variants	None coding variants	Amino acid changes
Male	97610	16623	614	96995	202
Female	17024	3024	190	16834	51

Numerous differential variants were produced in this investigation (Table S4). Logically, filtration is highly required for the classification of differential variants in order to have a better understanding about their functions by available annotations. For example, known variants annotation was used to identify the novel variants. CDS (coding region) annotation option was selected for the recognition of coding variants in order to find out which variants were located on coding and non-coding regions. Coding variants were collected for functional consequences and amino acid changes analysis. Therefore, reference genome, CDS, and mRNA annotations were used to analyze the amino acid changes. In addition, Standard genetic code was selected in CLC genomics Genomic Workbench (8.5.1) for amino acid change analysis.

Novel variants: variants were reported for the first time.

Coding variants: variants were located in coding regions.

Amino acid changes: variations can change the protein sequence.

Totally, more than 30 GO terms were reported for differential variants in females. Considering the molecular functions, the most frequency of GO terms belonged to 5′-3′ exodeoxyribonuclease activity and histone binding, respectively. Furthermore, candidate genes of GO terms were suggested including *DCLRE1C* and *ATAD2*. It was also found that considering cellular components, the mitochondrion was identified as the most important organelle for hypoxia condition differing between highland and lowland ecotypes.

Results of biological process analysis showed that most of the GO terms were related to the DNA repair processes including double-strand break repair and inter strand cross-link repair.

Results of gene ontology enrichment among males indicated that several candidate genes such as *TATDN1*, *POLQ* and *DCLRE1A* were associated with DNA repair. It was observed in the biological process analysis that there were several Go terms, including pericardium morphogenesis and thalamus development. There were also several biological pathways suggested for DNA repair and biosynthetic (e.g., DNA biosynthetic process; double-strand break repair; DNA repair; and inter strand cross-link repair). The NK T cell differentiation was reported as a biological pathway of immune response in the hypoxia condition. To see more results of gene ontology enrichments analyses in males and females, refer to the Tables 7 and 8.

**Table 7:** Gene ontology enrichments analysis for differential variants – Male birds.

GO	GO term	Description and frequency	Overlapping genes	Chr	Type
Molecular function	0004536	Deoxyribonuclease activity (5%)	*TATDN1* (*Asp190Glu*)	2	SNV
	0044822	Poly (A) RNA binding (9%)	*RARS2* (*Gln274Lys*)	3	SNV
	0003785	Actin monomer binding (5%)	*COBLL1*	7	SNV
	0003887	DNA-directed DNA polymerase activity (14%)	*POLQ* (*Thr514Ala*)	1	SNV
	0051575	5′-deoxyribose-5phosphate lyase activity (10%)			
	0015410	Manganese-transporting ATPase activity (9%)	*ATP2C1* (*Asp383Glu*)	2	SNV
	0034185	Apolipoprotein binding (5%)	*LRP6* (*Thr522Ser*)	1	SNV
	0005041	Low-density lipoprotein receptor activity (5%)			
	0035312	5′-3′ exodeoxyribonuclease activity (14%)	*DCLRE1A*	6	SNV
	0030553	cGMP binding (10%)	*CNGA3*	1	SNV
	0004582	Dolichyl - phosphate beta-D-mannosyltransferase activity (14%)	*ALG5*	1	SNV
Cellular component	0000799	Nuclear condensin complex (6%)	*NCAPD3*	24	SNV
	0005901	Caveola (9%)	*LRP6* (*Thr522Ser*)	1	SNV
	0000139	Golgi membrane (11%)	*GOSR1*	19	SNV
	0005801	Cis-Golgi network (6%)			
	0005887	Integral to plasma membrane (29%)	*SLC38A6*	5	MNV
	0070419	Nonhomologous end joining complex (9%)	*PRKDC*	2	SNV
	0005654	Nucleoplasm (12%)			
	0000784	Nuclear chromosome, Telomeric region (6%)	*DCLRE1A*	6	SNV
	0030686	90S preribosome (6%)	*UTP20*	1	SNV
	0070382	Exocytic vesicle (6%)	*SYTL2*	1	SNV
Biological process	0006303	Double-strand break repair via nonhomologous end joining (4%)	*DCLRE1A*	6	SNV
	0006874	Cellular calcium ion homeostasis (12%)	*ATP13A4* (*lu589Asp*)		
	0003344	Pericardium morphogenesis (6%)	*LRP6* (*Thr522Ser*)	1	SNV
	0021794	Thalamus development (5%)			
	0030901	Midbrain development (6%)			
	0021987	Cerebral cortex development (7%)			
	0060059	Embryonic retina morphogenesis in camera-type eye (4%)			
	0060325	Face morphogenesis (1%)			
	0071897	DNA biosynthetic process (1%)	*POLQ*	1	SNV
	0006302	Double-strand break repair (18%)			
	2000042	Negative regulation of double-strand break repair via homologous recombination (4%)			
	0001865	NK T cell differentiation (2%)	*BMX* (*Arg122Lys*)	1	SNV
	0006281	DNA repair (25%)	*PRKDC*	2	SNV
	0036297	Inter strand cross-link repair (4%)	*DCLRE1A*	6	SNV

**Table 8:** Gene ontology enrichments analysis for differential variants – Female birds.

GO	Go term	Description and frequency	Overlapping genes	Chr	Type
Molecular function	0005198	Structural molecule activity (7%)	*PSMD13* (*Ile118Thr*)	5	SNV
	0035312	5′-3′ exodeoxyribonuclease activity (14%)	*DCLRE1C*	1	Deletion
	0005049	Nuclear export signal receptor activity (3%)	*RANBP17*	13	SNV
	0008536	Ran GTPase binding (7%)			
	0042393	Histone binding (13%)	*ATAD2*	2	SNV
	0008453	Alanine-glyoxylate transaminase activity (14%)	*AGXT2* (*Ala436Gly*)	Z	SNV
	0030170	Pyridoxal phosphate binding (12%)			
	0004222	Metalloendopeptidase activity (9%)	*UQCRC1* (*Arg211Gln*)	12	SNV
	0050839	Cell adhesion molecule binding (8%)	*CD200* (*His135Asn*)	1	SNV
	0004872	Receptor activity (13%)			
Cellular component	0070545	PeBoW complex (3%)	*PES1*	15	Deletion
	0005750	Mitochondrial respiratory chain complex III (9%)	*UQCRC1* (*Arg211Gln*)	12	SNV
	0022624	Proteasome accessory complex (9%)	*PSMD13* (*Ile118Thr*)	5	SNV
	0008541	Proteasome regulatory particle (6%)			
	0070419	Nonhomologous end joining complex (12%)	*DCLRE1C*	1	Deletion
	0000784	Nuclear chromosome, telomeric region (13%)			
	0005769	Early endosome (2%)	*ZFYVE9*	8	SNV
	0031410	Cytoplasmic vesicle (17%)			
	0005840	Ribosome (12%)	*HSPA14* (*Leu397Va*)	1	SNV
	0005913	Cell-cell adherens junction (17%)	*CD200* (*His135Asn*)	1	SNV
Biological process	0006835	Dicarboxylic acid transport (3%)	*SLC13A2*	19	SNV
	0000463	Maturation of LSU-rRNA from tricistronic rRNA transcript (SSU-rRNA, 5.8S rRNA, LSU-rRNA) (7%)	*PES1*	15	Deletion
	0006611	Protein export from nucleus (8%)	*RANBP17*	13	SNV
	0006303	Double-strand break repair via non-homologous end joining (9%)	*DCLRE1C*	1	Deletion
	0036297	Inter strand cross-link repair (3%)			
	0031848	Protection from non-homologous end joining at telomere (4%)			
	0090305	Nucleic acid phosphodiester bond hydrolysis (15%)			
	0007156	Homophilic cell adhesion (8%)	*CD200 His135Asn*	1	SNV
	0007157	Heterophilic cell-cell adhesion (8%)			
	0008037	Cell recognition (5%)			
	0006511	Ubiquitin-dependent protein catabolic process (15%)	*PSMD13 Ile118Thr*	5	SNV
	0043248	Proteasome assembly (3%)			
	0009060	Aerobic respiration (9%)	*UQCRC1* (*Arg211Gln*)	12	SNV
	0006122	Mitochondrial electron transport, ubiquinol to cytochrome c (3%)			

Results of the GO analysis are given in Tables 7 and 8. The output of amino acid change analysis for differential variants was used for gene ontology enrichment analyses in three parts, including biological process, molecular function and cellular component. The gene ontology (GO) association file, which includes gene names and associated gene ontology terms, were downloaded from the gene ontology consortium (http://geneontology.org/) and imported to CLC Genomic Workbench (8.5.1). The significance level of GO analysis was also determined as 0.01.

### Identification of potential selective sweeps between the highland and lowland Chickens

The process of signature selection analysis (SSA) is carried out in this study in order to identify those genomic regions that were under the selection pressure. The main goal of SSA was to demonstrate that differential variants are actually adaptive in response to hypoxia and are not due to other evolutionary pressures or mechanisms of genetic changes.

**Figure 2:**
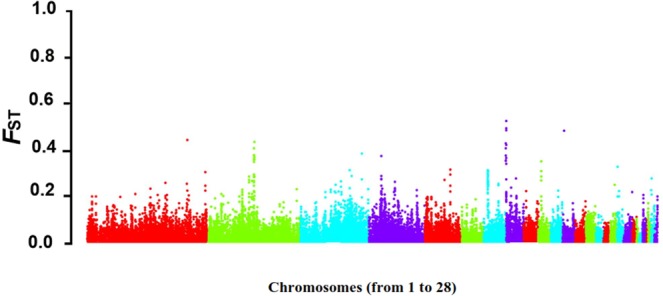
Genomic landscape of the population differentiation in highland and lowland chickens. In current study signature selection analysis was carried out for confirming the results of differential variant analysis. Therefore, for each population of highland and lowland chicken differentiation (F_ST_) was calculated for each SNV as described by Weir and Cockerham (1984) and sex chromosomes were removed. The average F_ST_ values of SNVs in each window were calculated and genomic landscape of the population differentiation between highland and lowland chicken was drawn by qqman package and R program (version 3.2.2). Based on extremely high Fst (top 5%) 95 biological categories and 858 candidate genes are reported to identify genomic regions which are under selection pressure.

According to the extremely high F_ST_ (top 5%), it was found that 95 biological categories and 858 candidate genes were reported from different databases including gene ontology consortium (GO), human phenotype ontology (HP), Kyoto encyclopedia of genes and genomes (KEEG), miRbase (mi), transcription factor database (TF) and the genomic landscape of the population differentiation (F_ST_) among highland and lowland chickens is shown in Fig. 2.

According to the results of SSA, it was found that several candidate genes were associated with the hypoxia condition. Several candidate genes including *MRE11A*, *DDIAS*, *PRIM2*, *DNASE*, and *MMS22L* were suggested as examples of the DNA response to UV radiation and DNA repair. More importantly, it should be noted that there were several biological categories enriched by SSA including immune response (*TRAT1*, *BMX*, *CXCR4*), protein hemostasis (*PSMD13*), and mitochondrion function (*COX7A2*, *MTO1*, *ME3*, *MICU3*, *HCCS*). Table 9 shows a summary of biological categories and candidate genes. Additionally, the results of SSA are completely provided in the Supplementary Dataset 11.

**Table 9:** The summary results of signature selection analysis (biological categories and candidate genes).

Type	ID	Category	Gene enrichments and functions	P-value
BP	GO:0044763	Single-organism cellular process	*MRE11A* (double-strand break repair protein) *MMS22L* (DNA repair protein) *TIMM10* (translocase of inner mitochondrial membrane 10 homolog)	0.03
CC	GO:0071944	Cell periphery	*TRAT1* (T cell receptor associated transmembrane adaptor 1) *CXCR4*(C-X-C chemokine receptor type 4)	0.4E-02
	GO:0005886	Plasma membrane	*BMX* (NK T cell differentiation)	
TF	TF:M00809_0	Factor: FOX;motif: KWTTGTTTRTTTW	*PRIM2* (DNA primase) *MRPS11*(28S ribosomal protein S11, mitochondrial) *MRPL46* (39S ribosomal protein L46, mitochondrial) *MELANOMA* (Gallus gallus mitochondrial ribosomal protein L28 (MRPL28), nuclear gene encoding mitochondrial protein)	0.2E-03
	TF:M00747_1	Factor: IRF-1; motif: TTCACTT	*DNASE2* (deoxyribonuclease-2-beta precursor)	0.2E-02
	TF:M03559_0	Factor: Pit-1; motif: NNWWATTCAT	*DDIAS* (Induced by UV radiation. DNA damage-induced apoptosis suppressor *DLD* (dihydrolipoyl dehydrogenase, mitochondrial) *HCCS* (cytochrome c-type heme lyase) *PSMG3* (proteasome assembly chaperone 3)	0.8E-06
	TF:M00432_0	Factor: TTF1; motif: ASTCAAGTRK	*BDNF* (Brain-derived neurotrophic factor)	0.3E-02
	TF:M00206_0	Factor: HNF-1; motif: DGTTAATKAWTNACCAM	COX7A2 (cytochrome c oxidase subunit 7A2) *MTO1* (protein MTO1 homolog, mitochondrial) *SERPINB10* (Heterochromatin-associated protein MENT. Chromatin DNA binding)	0.6E-02
	TF:M00410_0	Factor: SOX9; motif: NNNNAACAATRGNN	*ME3*(malic enzyme 3, NADP(+)-dependent, mitochondrial)	0.6E-02
	TF:M00042_0	Factor: SOX5; motif: NNAACAATNN	*DARS2* (aspartyl-tRNA synthetase 2, mitochondrial)	0.02
	TF:M01292_1	Factor: HOXA13; motif: ATAAMA	*PRELID1* (PRELI domain-containing protein 1, mitochondrial)	0.1E-06
mi	MI:bta-miR-22-5p	MI:bta-miR-22-5p	*MICU3* (mitochondrial calcium uptake family member 3)	0.04

In this study, signature selection analysis was carried out for confirming the results of differential variants analysis. Therefore, we can demonstrate that the differential variants are actually adaptive in response to hypoxia and are not due to other evolutionary pressures or mechanisms of genetic change. Based on our results, 95 biological categories and 858 candidate genes were reported by signature selection analysis. We found that the results of signature selection analysis are similar to differential variant analysis. For example, DNA repair (*MRE11A*, *MMS22L*) and immune response (*BMX*, *TRAT1*, *CXCR4*). The summary results of signature analysis are presented in Table 9. In this way, all results are prepared completely in supplementary dataset 11.

### Common variants between highland and lowland chickens

Generally, in both males and females, 53780 variants were shared between highland and lowland chickens; 17835 new variants were also reported for the first time. It was found that 516 out of 53780 variants could lead to changes in amino acid sequences (Supplementary Table S5).

Results of gene ontology enrichment analysis of detected variants, in females and males, indicated that GO terms were related to the cell survival. For example, cell proliferation, cell growth (*ROS1*), digestion (*PRSS3*), chromosome organization (*BRCA2*), RNA processing (*TDRD9*), telomere capping (*POT1*), and cytokinesis (*CEP55*). In order to see more details of common variants, refer to the Supplementary text and Supplementary Tables S6–S7.

### mtDNA variants

In general, for both highland and lowland chickens, 50 variants were detected in mtDNA (10 samples), and most of them were considered as the novel variants (Table 10). One significant gene ontology (GO term: 1902600) was observed in mtDNA variants. It was reported that, for highland chickens, hydrogen ion transmembrane transport was a biological pathway in males. It is involved in the directed movement of hydrogen ion (proton) across a membrane. Noticeably, *COX3* gene (cytochrome c oxidase III) was considered in this biological pathway.

**Table 10:** Classification of mtDNA variation in highland and lowland chickens

Population	SNV	Insertion	Replacement	Total	New variants	Coding variants	Amino acid changes
Highland-male	21	1	1	23	23	14	5
Highland-female	9	0	0	9	8	4	1
Lowland-male	8	0	0	8	8	6	2
Lowland-female	10	0	0	10	10	7	2

In this study, mtDNA variants were analyzed, separately. After variants calling, all mtDNA variants including SNV, insertion and replacement were obtained from highland and lowland chickens, separately. Filtrations were also carried out same as what described for differential variants. We recognized that highland chickens had more variants than lowland chickens (32 versus 18 variants). It might be explained that more variants in highland chickens were created in order to adapt to the high-altitude conditions.

Novel variants: variants were reported for the first time.

Coding variants: variants were located on coding regions.

Amino acid changes: The protein sequence can be changed by the variations.

### Validations of novel differential SNVs between highland and lowland ecotypes and mtDNA variants

Results of the validation indicated that 19 out of 32 novel variants were validated in mtDNA. Five variations of regions including 5928, 6758, 8070, 8330, 11378 had the most validating percentage of mtDNA (Table 11). Results of the validation of novel differential SNVs showed that 19 out of 3845 variants were validated in five new samples (Table 11).

**Table 11:** Validation of novel differential SNVs and mtDNA variants in highland chickens.

Chr	Region	Genotype	Validation %	Variant type
MtDNA	164	C/T	20	Single nucleotide variant (SNV)
	307	C/T	20	
	443	C/T	20	
	**5928**	C/A	**40**	
	**6758**	T/C	**40**	
	6800	T/C	20	
	7530	C/G	20	
	**8070**	T/C	**40**	
	9533	A/G	20	
	10072	A/G	20	
	11378	C/T	**40**	
	11963	C/T	20	
	16586	A/G	20	
	253	T/C	20	
	258	C/T	20	
	4580	G/A	20	
	**8330**	T/C	**40**	
	**11378**	C/T	**40**	
	12094	T/C	20	
1	1254679	A/G	20	
1	103830963	C/T	20	
**1**	**2704537**	**G/A**	**60**	
1	165925917	G/T	20	
1	89054158	C/T	20	
1	180719232	A/G	20	
1	138527429	G/A	20	
2	4877832	C/T	20	
2	56043229	C/T	20	
2	49059638	T/C	20	
3	33701810	A/G	20	
3	65865344	A/C	20	
4	76983077	A/G	20	
6	2014167	A/C	20	
7	17112008	C/G	20	
8	8248431	A/T	20	
9	2581777	C/T	20	
12	816375	A/G	20	
17	8030611	G/A	20	

Validations were performed for two groups of detected variants in this study. Frist, novel differential SNVs between highland and lowland chickens, and second, mtDNA variations in highland chickens. Thus, other five whole genomes of highland samples (Isfahan) were sequenced, separately. The whole genome sequencing, trimming and variant detection were carried out based on provided information in material and method sections. A total of 3845 SNVs was reported as new variants for differential variants analyses, and 32 variations were detected in mtDNA for highland samples. The region and chromosomes of each SNV were evaluated in the new five samples by R program (www.R-project.org) in order to validate the variants. The calculation of validation’s percentage was based on samples that had variants. Finally, 19 variants were validated for mtDNA and novel differential variants.

### The analysis of genomic variants enrichment for the differential variant between highland and lowland chickens

**Figure 3:**
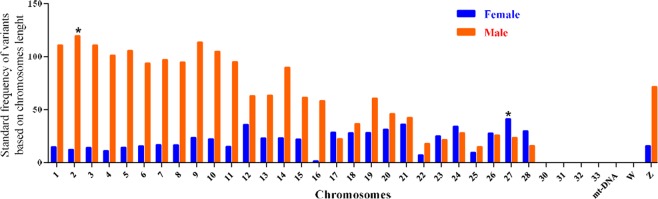
Standard frequency of differential variants between highland and lowland chicken for the selection of chromosomes that have the highest standard frequency. Dividing variants numbers by the chromosome length (Mb), standard frequencies of differential variants were estimated for each chromosome, then chromosomes with high-density of variants were selected (marked with asterisk) in order to be utilized for further analysis. Chromosomes 30–33 are not available in the genome reference.

**Figure 4:**
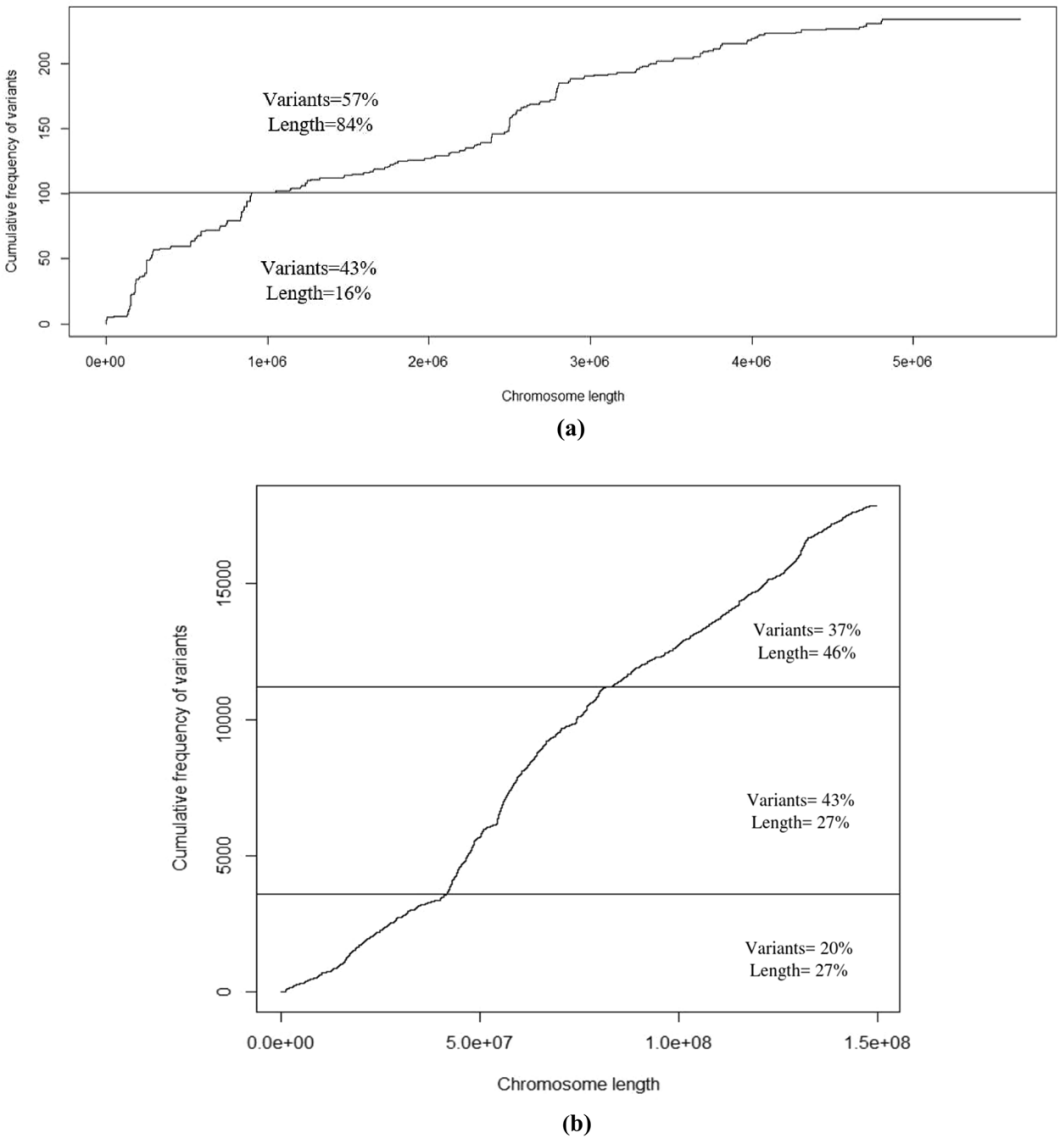
Cumulative frequency graph of variant numbers for chromosomes 27 (**a**) and 2 (**b**) in female and male chickens for genomic variant enrichment analysis. Chromosomes 2 and 27 (Fig. 2) were selected in order to draw the cumulative frequencies graph of variants along the chromosome. It is shown by Figure (**a**) that the most variants enrichments are available in the range of 2692–1 × 10^6^ base pairs (separated with horizontal line) of chromosome 27. A total of 234 variants was recognized on chromosome 27, and 100 (42.72%) variants were also located in the range of 2692–1 × 10^6^ region. Figure (**b**) indicates that 17891 variants were recognized on chromosome 2, but 7600 variants (43%) were located in the range of 41 × 10^6^–81 × 10^6^ (27% of chromosome length) base pairs. Finally, these regions with the highest density of variation were selected for the identification of candidate genes.

The chromosome that had the most standard frequency in females was known as the chromosome 27 (Fig. 3). In fact, 234 variants were recognized on chromosome 27; however, 100 variants (43% of variants) were located in the range of 2692–1 × 10^6^ base pairs (16% of the chromosome length) (Fig. 4a). Findings illustrated that 40 ENSGALT (Ensembl transcript) IDs were available in this region, and five Ensembl gene IDs were also detected by BioMart tools (Table 12). We found that MIR6644-2 gene contributed in the gastrulation. Gastrulation is recognized as one of the most important steps in animals’ embryonic development. Furthermore, the single layered blastula develops to a multilayered organization, called gastrula, during the gastrulation^17^.

**Table 12:** Genomic variants enrichment analysis for the identification of candidate gene associated with hypoxia in males and females.

Sex	Gene ID	Biotype	Function	Product name	Gene name	Uniprot ID
Female	ENSGALG00000028220	Protein coding	structural constituent of cytoskeleton	Keratin	*LOC107055272*	E1C3X3
	ENSGALG00000026257	Protein coding	structural constituent of cytoskeleton	Keratin	*LOC107055272*	E1BXQ5
	ENSGALG00000028852	miRNA	Gastrulation	MIR6644-2	*MIR6644-2*	No protein
	ENSGALG00000026447	Protein coding	Cytoskeleton organization	Keratin	*LOC428303*	E1C3N8
	ENSGALG00000027740	miRNA	Unknown	Unknown	Unknown	No protein
Male	ENSGALG00000011733	Protein coding	immune response	C-C chemokine receptor type 2	*CCR2*	F1NPK8
	ENSGALG00000011734	Protein coding	chemotaxis	C-C chemokine receptor 8 like	*CCR8L*	F1NPK7
	ENSGALG00000011735	Protein coding	chemokine receptor activity	chemokine XC receptor 1	*CCXCR1-L*	Q702H5
	ENSGALG00000011808	Protein coding	macrophage receptor activity	C-C chemokine receptor type 9	*CCR9*	F1NMB1
	ENSGALG00000012671	Protein coding	Hormone activity	Prolactin	*Prl*	A0A0B5L5F3
	ENSGALG00000012700	Protein coding	ubiquitin-protein transferase activity	Ring finger protein 182	*RNF182*	E1BZ35
	ENSGALG00000012702	Protein coding	Chromatin binding	Protein Jumonji	*JARID2*	F1NWZ0
	ENSGALG00000012732	Protein coding	protein phosphatase regulator activity	Phosphatase and actin regulator	*PHACTR1*	F1NWB1
	ENSGALG00000012748	Protein coding	Elongation of very long chain fatty acids protein 2	fatty acid elongase activity	*ELOVL2*	E1BYE9

Identification of the genomic regions, which had the highest density of differential variant between highland and lowland chickens, is considered as the main purpose of genomic variants enrichment analysis. The candidate genes located in the regions of genome, which had the highest density of variants, can be observed in this table. We found that the most important functions that can be considered in low oxygen conditions are Immune response, chromatin binding, and gastrulation. In addition, MIR6644-2 was reported as the candidate gene in order to develop the gastrulation process, and was not implicated in the previous investigations about high-altitude adaptation of chickens.

The most standard frequency of variants of chromosome 2 in males was estimated to be 119.60 (Fig. 3). A total of 17891 variants was detected on chromosome 2, but 7600 variants (43%) were located in the range of 41 × 10^6^–81 × 10^6^ (27% of chromosome length) base pairs; however, this region, which had a high density of variation, was selected for further analysis (Fig. 4b). It was shown by results of UCSC and the table browser analysis that 412 ENSGALT IDs were available for the selected region and consequently, nine Ensemble gene IDs were identified by BioMart tools (Table 12). We found that some functions were involved in the adaptation to hypoxia including chemotaxis, immune response, inflammatory response, and positive regulations of monocyte chemotaxis. Other results indicated that Jumonji protein might have an important role in the adaptation to hypoxia. Reviewing the studies, we found that Jumonji protein family would contribute in the wide range of chromatin regulation, genes expression, and signaling pathways^18^. While the Jumonji is known as the DNA binding and transcriptional repressor, this protein interacts with the Polycomb repressive complex 2 (*PRC2*) in humans, which plays a critical role in the regulation of gene expression during the embryonic development (www.ncbi.nlm.nih.gov/gene/3720). This study indicates that *PRL* gene can play a very important role in the condition of lack of oxygen. It has been illustrated that the *PRL* gene is associated with reproductive traits; specially egg production traits^19^. In fact, *PRL* gene is a multifunctional hormone that impacts on multiple physiological processes including cardiovascular system; however, they have been poorly described^20^.

### The possible gene network of differential variants

**Figure 5:**
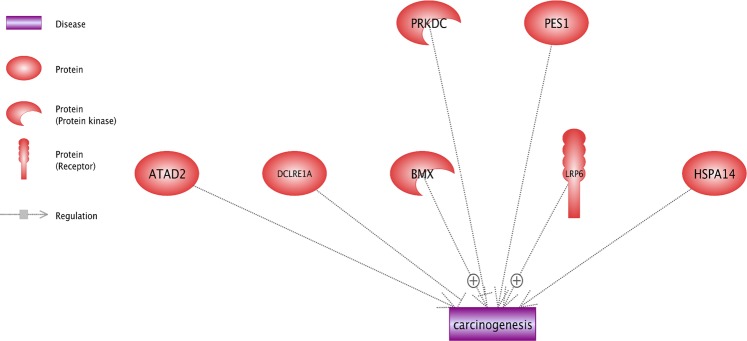
The network analysis for differential candidate gene in hypoxia condition for carcinogenesis. The high-dose UV radiation can lead to DNA damage and tumorigenesis in high-altitude condition; thus, gene networks analyses of key genes involved in the high-altitude adaptation could provide new information about carcinogenesis. A total of 27 candidate genes was identified for differential variants in males and females in this investigation (Tables 7 and 8). Chicken Ensembl gene identifications were converted into human orthologs in order to construct an informative network and finally, results showed that seven candidate genes were enriched in carcinogenesis network and had a regulatory role. Specifically, *BMX* and *LRP6* induce the carcinogenesis and *HSPA14*, and *DCLRE1A* inhibits that.

**Figure 6:**
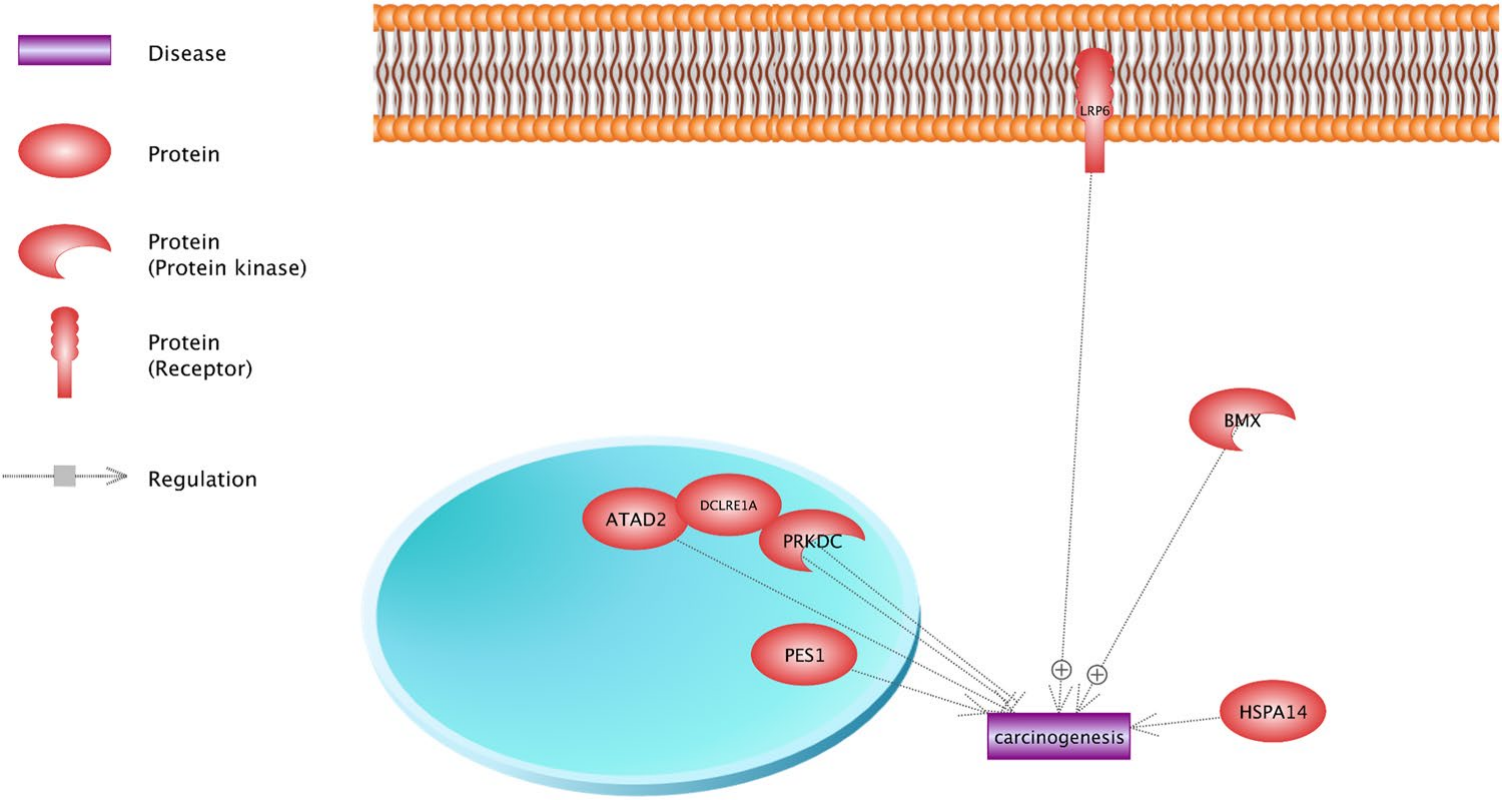
The cellular location of the key proteins in the regulation of carcinogenesis. This figure shows the cellular location of the key proteins in three parts of the cell (Membrane, Nucleus and Cytoplasm). *LRP6* is known as receptor, thus cellular location of this protein is shown in the membrane. Similarly, other proteins including *DCLRE1A* contribute in the DNA repair process. Consequently, it is illustrated in nucleus. These results can be useful for better understanding of the diseases creation process and the selection of appropriate drug design methods, based on cellular locations of key proteins in carcinogenesis.

Results of the analysis of gene network indicate that seven differential candidate genes contribute in the regulation of carcinogenesis. It is observed that most of the candidate genes induce carcinogenesis, especially *BMX* and *LRP6* (Fig. 5). However, *HSPA14* and *DCLRE1A* genes had an inhibition impact on the tumorigenesis. See the Supplementary Table S8 for more details about underlying references of relationships. Figure 6 shows the cellular location of candidate gene. For example, *LRP6* is considered as the receptor; therefore, it is shown in the membrane cell. In addition, *BMX* and *HSPA14* were activated in cytoplasm, but other candidate genes were in the nucleus. This information could be useful in order to explore the contribution of genes in biological pathways. For instance, *DCLRE1A* and *PRKDC* have a critical role in the process of DNA repair^21^. Therefore, their location can also be observed in nucleus. These results could be helpful in order to understand the process of diseases creation and select the appropriate drug design method, based on the cellular location of key proteins in carcinogenesis.

### The submission of novel variants to European Bioinformatics Information (EBI)

Many SNVs are detected as the novel variants in this investigation. Novel variants were submitted to EBI database with the accession number of PRJEB24944. See Table S9 for more details. Discovered variants can be utilized for future breeding programs. The analysis of data with hierarchical pattern recognition algorithms can be ranked, thus the best combination of variants distinguishing highland and lowland chickens will also be found^22,23^.

## Discussion

Full genome sequencing of highland and lowland chicken ecotypes are utilized in this investigation in order to unravel molecular mechanisms of adaptation to the condition of lack of oxygen. Recently, researchers are more willing to study the mtDNA variation and ATP production in low oxygen conditions. According to our findings, hydrogen ion transmembrane transports in mitochondrial inner membrane, as a biological pathway involves in mitochondrial respiratory, and *COX3* candidate gene (cytochrome c oxidase III or *MT-CO3*) was also detected for this biological pathway. It is the terminal enzyme of the respiratory chain of mitochondria, which is involved in the transfer of electrons from reduced cytochrome c to the molecular oxygen^24^. Results of this study corroborate the findings of Sun *et al*.^25^ who investigated the association between *MT-CO3* (mitochondrially encoded cytochrome c oxidase subunit III) gene and high-altitude adaptation in Tibetan chickens. They demonstrated that eight SNPs were available (single nucleotide polymorphisms), and five of them were shared by Tibetan chickens and lowland chickens.

Results showed that highland chickens had more mtDNA variants than lowland chickens (32 versus 18 variants). It is a reasonable approach to consider that more variants in highland chickens were created in order to be adapted to high altitude conditions. Amazingly, there is a severe sex bias in mitochondrial nucleotide variation, as male highland chickens had more variants than female highland chicken (a ratio of almost 3:1). A possible explanation for this might be that female and male chickens are different in sexual traits such as, fertility, gaining and consequently, energy consumption. In this way, Camus *et al*.^26^ indicated that mtDNA variation can affect the regulation of healthy traits and contribute in sex-related differences. Furthermore, the interactions between mtDNA and nuclear DNA involved in sex-specific transcript responses^27^.

In addition, we have detected novel mtDNA variations for the first time. Thus, our findings provided new potential molecular genetics for the genetic adaptation to high-altitude conditions. Logically, further examinations will be required in order to describe the main role of novel variants in low oxygen conditions.

**Figure 7:**
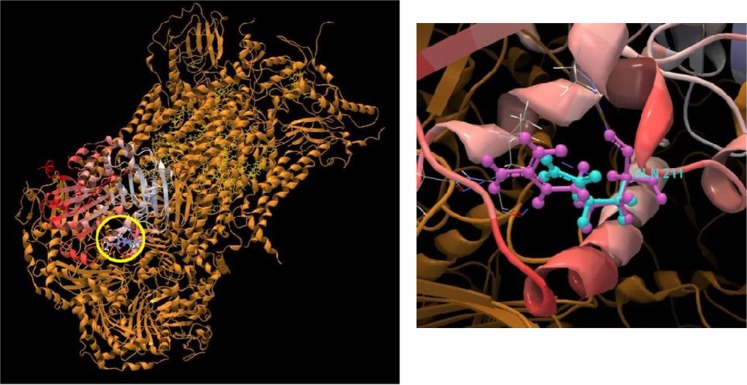
*UQCRC1* gene encodes ubiquinol cytochrome c reductase or cytochrome bc1 complex protein. It is a complex protein which is located on the mitochondrial inner membrane and involves in the cell respiration. The variants locations are shown by the yellow circle in the whole structure. Purple and green atoms show the reference and variants structures, respectively.

We found that there is a non-mitochondrial variant (chromosome 12), which is involved in the cell respiration. Mitochondrial respiratory complex chain III contributes in the ATP synthesis, electron transport, and metabolism. It is a complex protein located in the mitochondrial inner membrane^28^. Here, *UQCRC1* gene was reported in order to encode ubiquinol cytochrome c reductase or cytochrome bc1 complex protein. This might be explained by the fact that the activity of cytochrome c oxidase will decrease in the low oxygen condition and will also lead to the less activity of bc1 compound. Logically, the generation of reduced ubiquinol will be decreased^29^. Figure 7 shows the protein structure of *UQCRC1*.

Additionally, the proteasomes and protein translation tools are identified as interesting results of the cellular components analysis. There are evidence suggesting an association between hypoxic condition and protein hemostasis. Low oxygen condition affects the gene expression profiles in cell, in this way, transcription and translation process are changeable. Also, protein synthesis is one of the most energy-consuming process in cell related to normal oxygen concentration^30,31^. Thereby, cell reduces the messenger RNA (mRNA) rate to save energy metabolism while continuing the production of the proteins associated with survival factors^32^.

Gene ontology enrichment and genomic variants enrichment analysis, including immune response, histone banding, and organ development, also achieved the same results.

**Figure 8:**
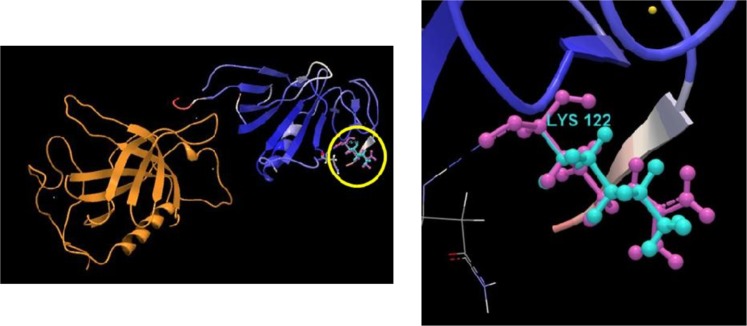
*BMX* gene encodes cytoplasmic tyrosine-protein kinase and contributes in the NK T cell differentiation. It plays a critical role in the induced interleukin-6 (IL6), adaptation of different cell systems against stress, growth, and differentiation of hematopoietic cell. Therefore, *BMX* gene was considered as the immune response in hypoxia conditions. The variants locations are shown by the yellow circle in the whole structure. Purple and green atoms also show the reference and variants structures, respectively.

Direct evidences were found on the association between solar radiation and immune response about the hypoxia condition in this investigation. Thus, it was recognized that NK (natural killer) T cell differentiation was considered as the immune response. *BMX* gene contributes in the NK T cell differentiation. *BMX* gene encodes cytoplasmic tyrosine-protein kinase in human being^33^. It plays a critical role in the induced interleukin-6 (IL6), adaptation of different cell systems against stress, growth, and differentiation of hematopoietic cells^34^. Findings of this study are consistent with those of McNamee *et al*.^35^, which indicate that the differentiation of T cells could be stimulated by hypoxia and high altitude conditions. Also, Zhang *et al*.^15^ showed that there is an association between the high altitude condition and inflammation responses. Protein structures and the variant location of *BMX* are shown in Fig. 8. Similarly, results of the analysis of genomic variants enrichment indicated that chemokine receptors have a critical role in the immune response. Chemokines are defined as small protein molecules produced by cells of the immune system. They contribute in the migration of immune cells to an infection site. Furthermore, chemokine receptors are involved in monocyte infiltration of inflammatory responses against tumors (Uniprot: Q8QG57).

Several studies indicated that epigenetic regulations in cells might be caused by the hypoxia^36–38^. Results of gene ontology showed that histone binding might be associated with the hypoxia condition. Furthermore, we suggested that *ATAD2* gene can be considered for the histone binding process. *ATAD2* gene belongs to the ATPase family and is involved in the chromatin binding and histone binding (UniProt: Q6PL18). This finding is in accordance with that of Zhang *et al*.^15^, which indicated that there was an association between histone binding and high altitude conditions. In addition, the analysis of genomic variants enrichment confirmed that Jumonji protein and *JARID2* can contribute in the hypoxia adaptation process by chromatin bindings. It has been demonstrated that, *JARID2* gene plays an essential role in embryonic and organ developments including heart development, and neural tube fusion (UniProt: Q92833).

**Figure 9:**
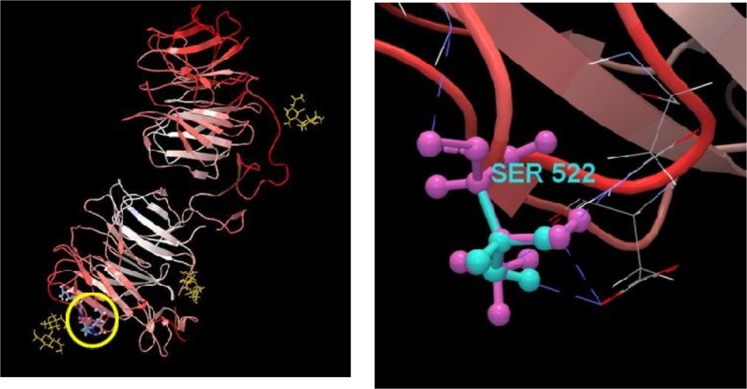
*LRP6* gene was considered as a candidate gene for organs development in high altitude conditions. *LRP6* gene encodes low-density lipoprotein receptor-related protein 6 and is a key regulator of tissue development and hemostasis. The yellow circle in the whole structure shows variants locations. The reference and variants structures are also shown by purple and green atoms, respectively.

Other interesting findings of the gene ontology enrichment analysis are considered as organ developments including brain development and pericardium morphogenesis. A strong relationship between high altitude condition and organs development has been reported by Zhang and Burggren^39^. For instance, highland chickens have smaller organs including heart, liver and lungs^15^. Our study presents evidences in order to prove that brain development has a critical role in the adaptation to high altitude conditions. It may be explained by the fact that brain is the most sensitive organ to the lack of oxygen, thus cerebral anoxia induces neuronal cell death and apoptosis and will also lead to hypoxic brain injury^40^. We found that there are gene ontology terms which impact on the brain development in highland chickens including mid brain development, cerebral cortex development, and thalamus development. It is recognized that pericardium morphogenesis was effective in high altitude conditions. Similarly, Patterson and Zhang^41^ demonstrated that normal heart formation and maturation in fetus depend on low oxygen stress, but further abnormal low oxygen condition has a negative influence on the structure, function, and gene expression in the fetal heart tissue. This is the evidence which is in accordance with our results. Therefore, *LRP6* gene was considered as a candidate gene for organs development in high altitude conditions. *LRP6* gene encodes low-density lipoprotein receptor-related protein 6 that has included co-receptor groups in the canonical Wnt pathway (Wnt/β-catenin pathway). It is considered as a very important regulator of tissue development and hemostasis^42^. Figure 9 shows the protein structure and variant location of *LRP6*.

Other interesting GO terms contained in high-altitude adaptation pathways- including DNA repair, double strand break repair, DNA biosynthetic process and inter strand cross-link repair activity- can be found in this study. One possible explanation might be defined as: high-dose UV radiation can lead to DNA damage, cell apoptosis, skin cancer and tissue injury of mammals^43,44^ in high-altitude conditions. In this study, we reported *DCLRE1C* gene as a candidate gene for DNA repair process. In humans, *DCLRE1C* gene encodes a single-strand nuclear protein that has a specific 5′-3′ exonuclease activity. Interestingly, this protein has main functions in the regulation of the cell cycle in response to DNA damage^21^.

Amazingly, it should be noted that comparable results were obtained by SSA and differential variants analysis. The results of differential variants analysis indicated that adaptive variants were involved in processes of DNA repair, organs development, immune response, epigenetic regulation, mitochondrion function (cell respiration), and protein hemostasis. Interestingly, similar results were reported by SSA and it is also recognized that both analysis show the similar gene’s functions that contribute in response to hypoxia. For instance, cell respiration and mitochondrion function were considered as the most important results of differential variants analysis in low oxygen condition. Thus, *UQCRC1* (mitochondrial respiratory chain complex III) was suggested as the candidate gene of cell respiration. Also, several candidate genes associated with cell respiration and mitochondrion function, including *COX7A2*, *ME3* and *HCC*, were reported by SSA. According to the results of SSA, it is recognized that adaptive variants in differential variant analysis are due to the adaptation process. Table 13 shows a summary of the comparison of results of SSA and differential variants analysis.

**Table 13:** The comparison results of signature selection and differential variant analysis.

Categories	Gene name	Description	SSA	DVA
DNA repair	MMS22L	DNA repair protein	*	
	PRIM2	DNA primase	*	
	MRE11A	Double-strand break repair protein	*	
	DNASE2	Deoxyribonuclease-2-beta precursor	*	
	DDIAS	Induced by UV radiation. DNA damage-induced apoptosis suppressor	*	
	DCLRE1A	5′-3′ exodeoxyribonuclease activity		*
	TATDN1	Deoxyribonuclease activity		*
	DCLRE1A	Double-strand break repair		*
	COBLL1	DNA biosynthetic process		*
	POLQ	Double-strand break repair		*
	PRKDC	DNA repair		*
	DCLRE1A	Inter strand cross-link repair		*
Immune response	TRAT1	T cell receptor associated transmembrane adaptor 1	*	*
	BMX	BMX non-receptor tyrosine kinase	*	
	BMX	NK T cell differentiation		*
	CXCR4	C-X-C chemokine receptor type 4	*	
Organ development	BDNF	Brain-derived neurotrophic factor	*	
	LRP6	Cerebral cortex development		*
	LRP6	Midbrain development		*
	LRP6	Thalamus development		*
Mitochondrion function (cell respiration)	COX7A2	Cytochrome c oxidase subunit 7A2	*	
	MTO1	Protein MTO1 homolog, mitochondrial	*	
	ME3	Malic enzyme 3, mitochondrial	*	
	DARS2	aspartyl-tRNA synthetase 2, mitochondrial	*	
	PRELID1	PRELI domain-containing protein 1, mitochondrial	*	
	TIMM10	Translocase of inner mitochondrial membrane	*	
	MRPS11	28S ribosomal protein S11, mitochondrial	*	
	MRPL46	39S ribosomal protein L46, mitochondrial	*	
	MELANOMA	Gallus gallus mitochondrial ribosomal nuclear gene encoding mitochondrial protein	*	
	DLD	dihydrolipoyl dehydrogenase, mitochondrial	*	
	MICU3	Mitochondrial calcium uptake family ember 3	*	
	HCCS	Cytochrome c-type hemelyase	*	
	UQCRC1	Mitochondrial electron transport		*
	UQCRC1	Mitochondrial respiratory chain complex III		*
Epigenetic regulation	ATAD2	Histone binding		*
	SERPINB10	Heterochromatin-associated protein MENT. Chromatin DNA binding	*	
Protein hemostasis	PSMD13	Proteasome accessory complex		*
	PSMD13	Proteasome regulatory particle		*
	PSMD13	Proteasome assembly		*
	PSMG3	Proteasome assembly chaperone 3	*	

The comparable results were obtained by signature selection and differential variants analysis. The results of differential variants analysis indicated that adaptive variants were involved in DNA repair, organs development, immune response, epigenetic regulation, mitochondrion function (cell respiration) and protein hemostasis. Interestingly, the similar results were reported by signature selection analysis and it is recognized that both analysis show the similar gene’s functions that contribute in response to hypoxia. SSA: signature selection analysis. DVA: differential variant analysis.

Results of the analysis of genomic variants enrichment showed that miRNAs can be considered in the high-altitude adaptation. Clearly, miRNAs involve in the regulation of different pathways in various cell types; therefore, they can provide new evidence about hypoxia-related gene regulations^45^. Several studies showed that an association between hypoxia and hypoxia-inducible factor (HIF) transcription factor is available, and will lead to the expression of a different type of genes such as miRNAs^46,47^. It was recognized that MIR6644-2 was reported as the candidate gene in the high-altitude adaptation process by our genomic variants enrichment analysis. Despite there was no previous study about MIR6644-2 gene and high-altitude adaptation; however, this has been proved that it has a main role in the gastrulation^48^. This is recognized as one of the most interesting discussions about hypoxia and gastrulation, that chronic hypoxia alters the physiological and morphological pathways of developing animal embryos^49,50^.

In this study, we carried out the gene network analysis in order to identify that candidate genes were enriched in human diseases. Although the biological pathways in animals are not in a good accordance with human being, there are many treatment systems and drugs developed in animal models, especially chicken models for human genetic and disease^51–53^. We found that the gene network was associated with the carcinogenesis. This result may be explained by the fact that, UV radiation in highlands leads to the DNA damage. Similarly, there is an association between cancers and DNA damage. Interestingly, the network analysis indicated that *DCLRE1C* and *PRKDC* proteins can inhibit the tumorigenesis. It is because the *DCLRE1C* and *PRKDC* proteins involve in DNA repair process^21^. In addition, *PRKDC* protein has a critical role in the development of immune system by maturing B and T cells^54^. This finding supports the previous research which showed the DNA-PKcs-deficient (encoded by *PRKDC* gene) associated with immune deficient, radio sensitivity and tumor development in mice^54,55^. We found that *PES1*would induce tumorigenesis. It is a possible explanation that *PES1* encodes pescadillo ribosomal biogenesis factor 1, which is a member of the PeBoW complex and has a main role in regulating the cell cycle. Therefore, the abnormal mitosis and carcinogenesis might be caused by the disruption of PeBoW complex. These findings further support the idea that *PES1* may involve in the promotion of the malignant phenotypes of colon cancer cells and the gastric cancer^56,57^. Also, it was found that *LRP6* would lead to the cancer disease. Cell receptors are considered as biomarkers in cancer researches^58^. There are several studies attempt to explain the role of *LRP6* receptor in tumorigenesis including triple negative breast cancer and human hepatocellular carcinoma^59–63^. Considering the Wnt signaling pathway (Wnt/β-catenin pathway) and normal cell cycle regulation, *LRP6* is a member of the LDL receptor family^63^. *HSPA14* was also reported as another candidate gene in carcinogenesis, and *HSPA14* is recognized as the heat shock protein family (member 14). Also, it is known as the HSP70-4 or HSP70L1. Previous studies indicated that *HSPs* involves in the wide range of tumor^64–67^. Similarly, Rerole.et.al.^68^ showed that *HSP70* are expressed abnormally as well as it causes tumor growth and metastasis in rodents.

See the Supplementary text for more information about the discussion part.

## Conclusions

This is a comparative genomic study of Iranian native chicken ecotypes in order to identify the genomic region associated with hypoxia and low oxygen condition. The detection of candidate genes in this study would provide new insights about hypoxia. These adaptive genes were related to important functions including DNA repair, organ development, immune response, and histone binding. The comparable results were obtained by signature selection and differential variant analysis, in this way we demonstrate that the differential variants are actually adaptive in response to hypoxia and are not due to other evolutionary pressures or mechanisms of genetic change.

We have also found that variations in mtDNA, including *COX3* gene, play an important role in the process of adaptation. It was identified that several candidate genes were associated with carcinogenesis. This is the first investigation that sequences the whole genomes of highland and lowland native chickens; therefore, many novel variants are discovered and submitted to EBI (PRJEB24944). Moreover, MIR6644-2 gene is considered as a candidate gene which might impact on the adaptation process to hypoxia by the regulation of embryo development, and was not implicated in previous investigations of high-altitude adaptation among chickens.

Therefore, this paper can provide new information about the adaptation process. The signature of native chicken, which ecotypes to high altitude and low oxygen at the genomic level, is produced for better understanding about hypoxia adaptation mechanisms.

## Materials and Methods

**Figure 10:**
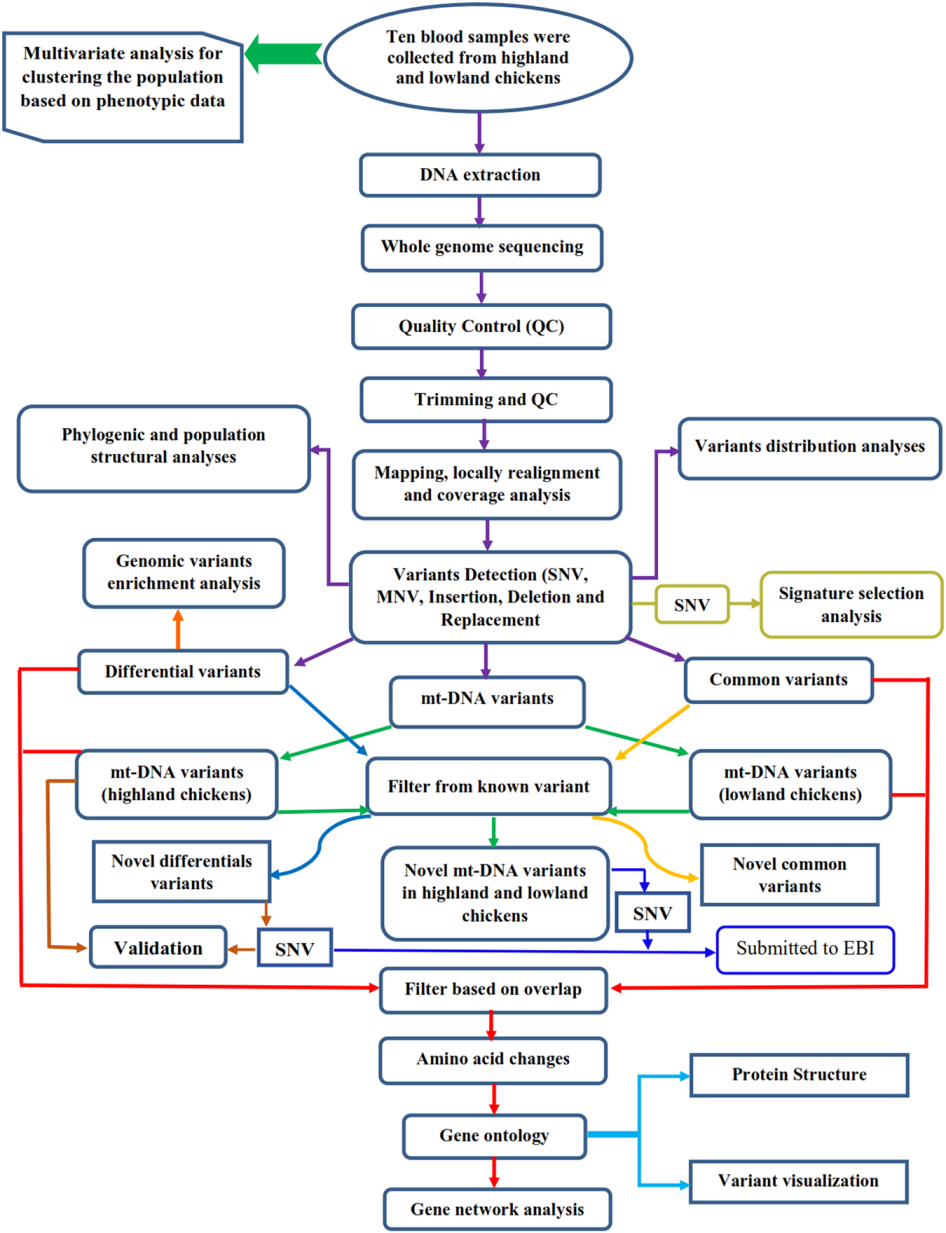
The Summary of sampling and data analysis for whole-genome sequencing of native chicken ecotypes and variant discovery associated with high-altitude adaptation (the same colors of vectors show the similar analysis).

The summary of sampling and whole genome data analyses are depicted in Fig. 10.

### Ethical approval statements

This investigation is in accordance with relevant guidelines and regulations of Shiraz University. All experimental protocols were approved by Institute of Biotechnology at Shiraz University.

The whole procedure of blood sampling was approved by the Department of Animal Science at Shiraz University (Permit number: 93-192). No birds were slaughtered and harmed.

### Blood sampling and DNA extractions

**Figure 11:**
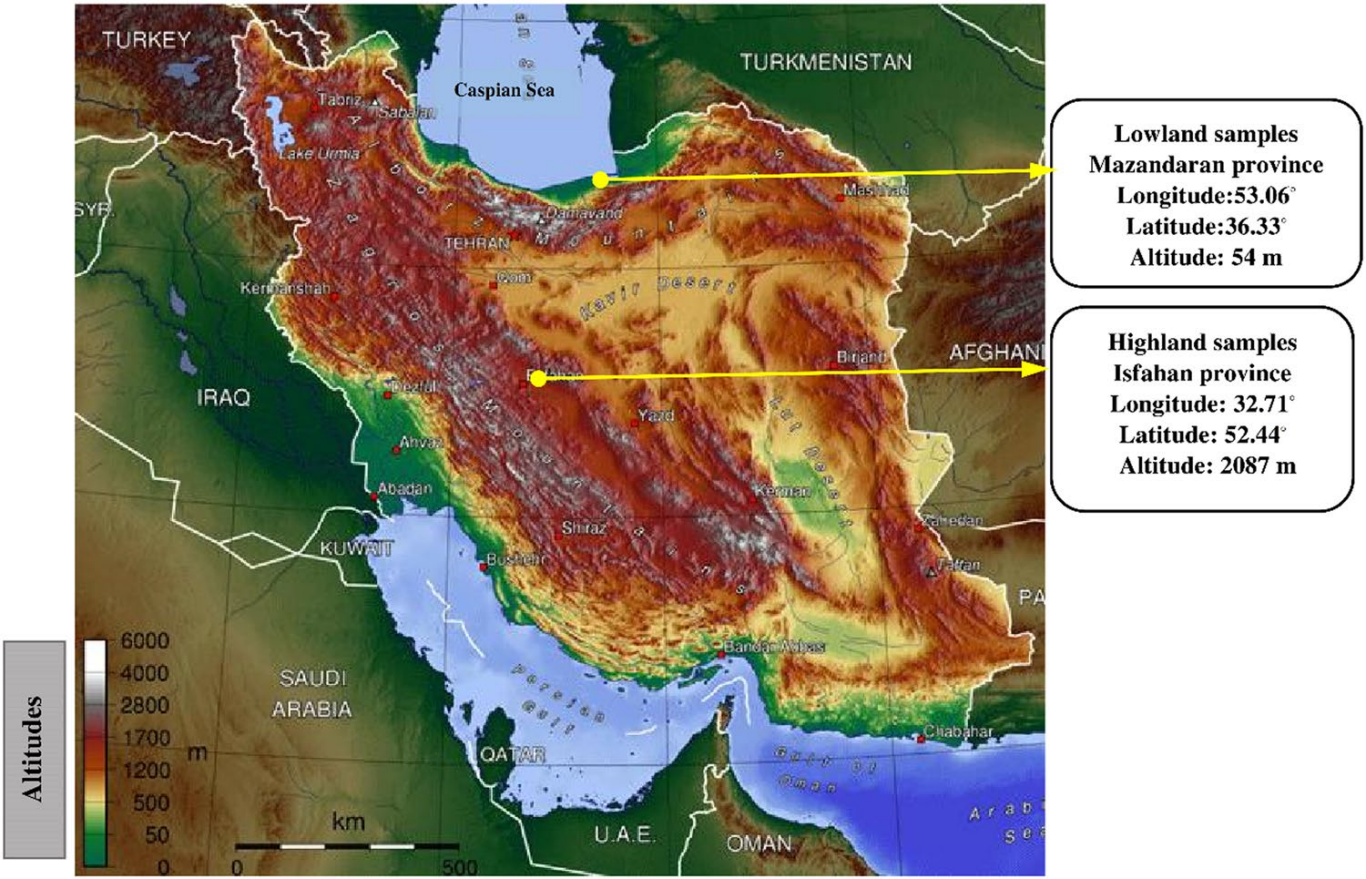
Sampling locations of highland and lowland chickens in Isfahan and Mazandaran provinces, located at different altitudes in Iran. Ten blood samples, including three males and two females from highland and two males and three females from lowland, were collected in order to be utilized in the whole genome sequencing. This figure was downloaded from an open source website https://commons.wikimedia.org. The direct URL is https://upload.wikimedia.org/wikipedia/commons/9/99/Iran_topo_en.jpg. The link to the license is https://creativecommons.org/licenses/by-sa/4.0/deed.en. Outer areas from the original figure were cropped, therefore “Caspian Sea” was rewritten in the above of figure based on the information of original figure. In addition, the locations of sampling were added to the figure by yellow vectors.

Blood samples were collected from Isfahan (highland, altitude = 2087 m) and Mazandaran (lowland, altitude = 54 m) provinces. Ten samples including three males and two females of the highland, and two males and three females of the lowland were collected. See Fig. 11 for more details about sampling locations. Two mL of blood was obtained from the birds’ wing vein. Using salting out protocol, the total DNA was isolated from the whole blood^69^. The process of testing DNA samples’ quality was carried out by the agarose gel (2%) and NanoDrop spectrophotometer, and high-quality DNA samples were also utilized for the subsequent whole genome resequencing.

### Phenotypic data and statistical analysis

Classification of highland and lowland chickens, based on phenotypic traits, was the main purpose of the statistical analysis. Twenty-four quantitative traits were recorded for 16 chickens of highland and lowland. To reduce measurement error, all chickens were matured while recording. The same recording protocol was also used in order to measure phenotypic traits of all the birds. Traits included the body weight (gr), neck length, body size (between waist and pectoral circumference), shank length, body size (between waist and abdominal circumference), wing length, tail length, femur length, crown length, crown height, back cape length, body size (between pectoral and cloaca circumference), head height (height from head to floor), wings length (open wings), number of toes, number of spur (nail of the foot), beak length, head circumference with a crown and a double wattles, toes length (right, middle, left), wattle height, wattle width, pectoral width, femur diameter and shank diameter. Utilizing the principle component analysis and discriminate analysis in Minitab software, we analyzed these traits (version: 17)^70^.

### Whole genome sequencing

The construction of genomic libraries was based on the Illumina standard genome library preparation pipeline. Using Hiseq. 2000 platform, whole genomes were sequenced and the length of provided paired-end short reads was also125 bp.

### Quality control and trimming

The function of quality control in CLC Genomic Workbench (8.5.1)^71^ was used by the following parameters for each sample: length distribution, GC content, ambiguous base content, Phred score, nucleotide contribution, enrich 5 mers, and duplicate sequences^72^. The adaptor sequences were removed by Illumina Company; thereby, trimming was carried out based on other parameters.

### Reference genome, mapping, locally realignments and coverage analysis

The reference genome and annotations were downloaded from the Ensembl database. Annotations included gene annotations and variants (ftp://ftp.ensembl.org/pub/relase-84/fasta/gallus_gallus). Mapping was carried out in CLC Genomics Workbench (8.5.1)^71^ based on the following parameters: masking mode = no masking, mismatch cost = 2, cost of insertions and deletions = linear gap cost, insertion cost = 3, deletion cost = 3, length fraction = 0.7, similarity fraction = 0.8, global alignment = no, Auto-detect paired distances = yes, and non-specific match handling = ignore. Finally, local realignment was used according to the following parameters: realign-unaligned ends = yes and multi-pass realignment = 2^73^.

The analysis of coverage was also utilized in order to identify the frequency of a base’s coverage in the reference genome by the mapped read of a given sequencing^74^.

Here, the analyses of coverage were estimated for all highland and lowland samples before and after the trimming process. Also, Lander/Waterman equation was used for the calculation of the coverage^75^.

$${\rm{C}}=\,\mathrm{LN}/{\rm{G}}$$

where, C stands for coverage, G is the haploid genome length (bp), L is the read length and N is the number of reads.

### Variant calling and statistical analysis

The variant detections algorithm was used by CLC genomics workbench (8.5.1)^71^. First, parameters were optimized for variant calling; therefore, some parameters were changed and tested for the optimization. Decreasing false variants was the main purpose of optimization (Supplementary Table S1). The ploidy level is fixed in chickens (2n = 78). Therefore, fixed ploidy algorithm^76^ was used for variant calling based on following parameters: required variant probability (%) = 95.0, ignore positions with coverage above = 50,000, restrict calling to target regions = not set, ignore broken pairs = yes, ignore non-specific matches = reads, minimum coverage = 10, minimum count = 2, minimum frequency (%) = 30, base quality filter = Yes, neighborhood radius = 15, minimum central quality = 30, minimum neighborhood quality = 25, remove pyro-error variants = yes, in homo-polymer regions with minimum length = 3-with frequency below = 0.8^72,76^.

We used the IDEG6 website for the statistical analysis^77^. Using Fisher exact test, we analyzed differences in variants distribution. The statistical analysis was carried out for each variant between highland and lowland samples, separately. Also, Chi-square test was established in order to analyze the differential distributed variations between highland and lowland chickens for each chromosome incorporating the sex of birds in the statistical analysis.

### Phylogenetic and structural population analysis

Any change in mitochondrial DNA (mtDNA) can be utilized as the phylogenic analysis, because mtDNA are considered as the conserved sequences. A total of 48 SNVs (single nucleotide variations) was identified in mtDNA using CLC Genomic Workbench (8.5.1)^71^, 480 SNVs were also genotyped (see results part) for ten samples. The phylogenic analysis was carried out based on 480 SNVs genotypes, Nei’s genetic distance matrix, and UPGMA method by PopGene software (version 1.32)^78^.

The population structural analysis was performed using multiple correspondence analysis (MCA) by R program (version: 3.2.2)^79^ and the “Ca” package^80^) in order to classify the highland and lowland chickens. We used the multiple correspondence analyses for qualitative data, while the principal component analysis (PCA) was used for quantitative data. MCA is another tool of multivariate methods that allows the analysis of systematic patterns of variations with categorical data^81^. 1000 SNVs genotypes were collected randomly from each highland and lowland chickens and a total of 10000 SNVs genotypes was used for MCA analysis.

### Differential variants

#### Comparing variants, filter variants and gene ontology enrichment analysis

After variants calling, variations of highland chickens were compared against the reads of lowland chickens as a control tool in order to remove the common variation between lowland and highland samples. Also, a comparison was carried out between male and female birds of highland and lowland, separately. We considered five frequency threshold percentages (0, 25, 50, 75 and 100) in order to be utilized for the frequency threshold optimization. The threshold frequency is considered as the percentage of samples that have variants. For instance, determining it to 50% indicates that at least 50% of samples, which were selected as the inputs, must contain a given variant in order to be reported in the output.

The differential variants were filtered based on known variants for the identification of novel variants. CDS (coding region) annotation was selected for overlap comparisons in order to understand which variants were located on coding and non-coding regions. Coding variants were collected for functional consequences and amino acid changes analysis. Reference genome, CDS and mRNA annotations were used for the amino acid change analysis. In addition, synonymous variants and CDS regions that had no variants were also filtered. In order to carry out the amino acid change analysis, standard genetic code was selected in CLC genomics^82^. The file of gene ontology (GO) association, which includes the gene names and associated gene ontology terms, was downloaded from the gene ontology consortium (http://geneontology.org/) and imported to CLC Genomic Workbench (8.5.1)^71^. The output of amino acid change analysis was utilized for the analyses of gene ontology enrichment of the biological process, molecular function, and cellular component. The significance level of GO analysis was determined to be 0.01.

#### Signature selection analysis

It should be noted that SSA was carried out in order to confirm the results of differential variant analysis in the current study. Considering descriptions of Weir and Cockerham^83^, (F_ST_) was calculated for SNVs of each population of highland and lowland chicken’s differentiation and sex chromosomes were removed. Sliding window analyses were carried out with a window size of 100 kb and a step size of 50 kb. Consequently, average F_ST_ values of SNVs in each window were calculated; also, the genomic landscape of the population differentiation between highland and lowland chicken was drawn by qqman pakage and R program (version 3.2.2)^79^.

Windows with the high 5% F_ST_ values were retrieved by using the Variant Effect Predictor online tool (http://www.ensembl.org). Also, the process of gene enrichment analysis was carried out by using g: profiler (http://biit.cs.ut.ee/gprofiler/index.cgi,).

Benjamini–Hochberg FDR (false discovery rate) was used in order to correct P values (Ming-Shan *et al*., Nosrati *et al*.)^84,85^. The summary of SSA are depicted in the online Supplementary Fig. S1. Finally, it should be noted that as it was previously described in CLC Genomic Workbench (8.5.1), all parameters of SNV detection existed in the command line tools were determined^71^.

#### Common variants discovery between highland and lowland chickens

**Figure 12:**
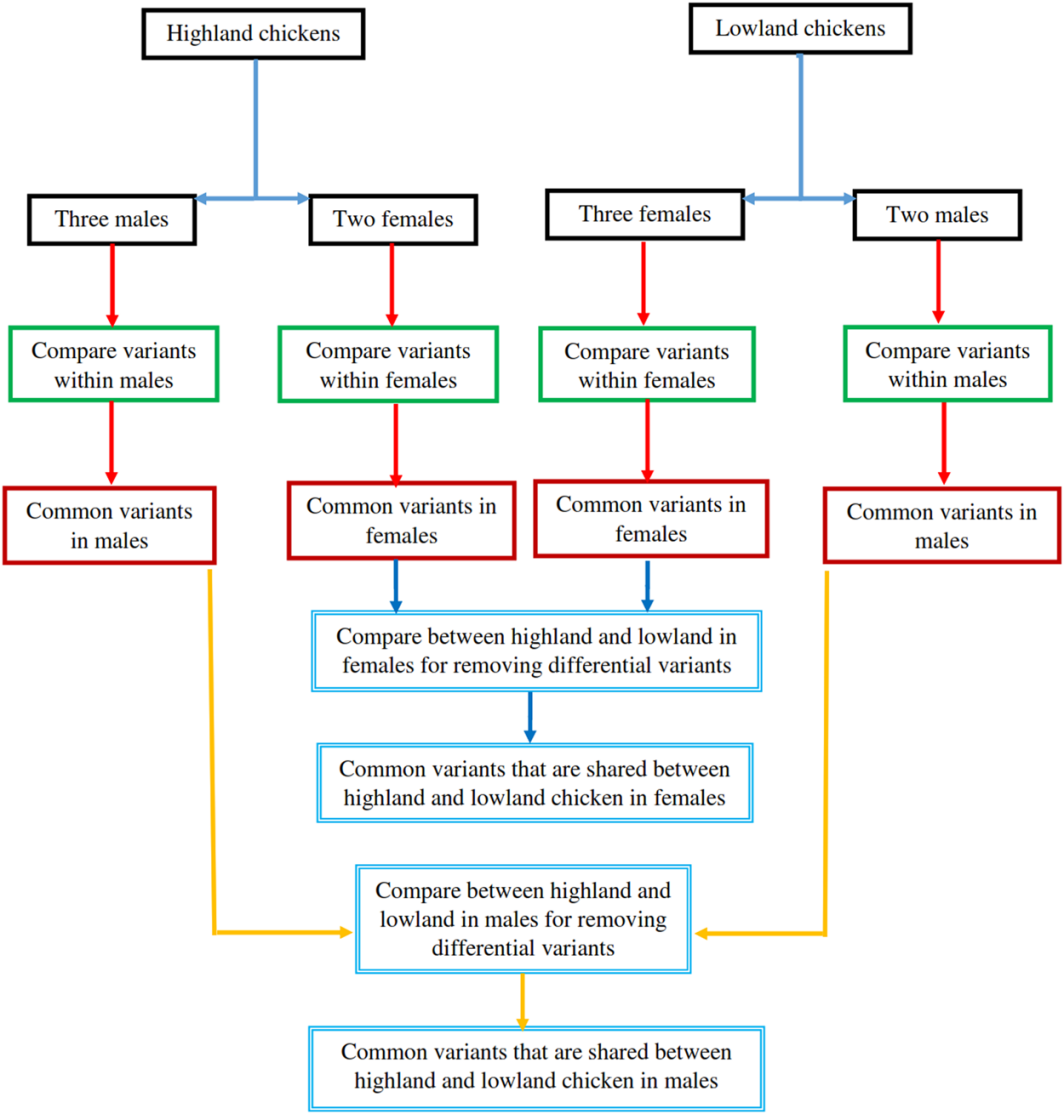
The process of common variants detection in highland and lowland native chicken ecotypes. This figure shows that how differential variants were removed between highland and lowland chickens. All comparisons of native chicken ecotype were carried out based on the sex of birds in each step. The frequency threshold was determined to be 100 percent in order to collect common variants between highland and lowland chickens.

The detection of common variants, shared between highland and lowland chickens is the main purpose of common variants discovery. See Fig. 12 for more details about common variants detection. 100% was determined for the frequency threshold. In the other words, all differential variants were removed completely, and common variants were kept for further analysis. The process of filtering and gene ontology enrichment analyses was same as what described for differential variants.

#### mtDNA variants

Ignoring the mtDNA variants in discovery variant projects is very difficult. The analyses of mtDNA variants were carried out separately in this investigation. After variants detection, all mtDNA variants were obtained in highland and lowland chickens, separately. Filtrations and gene ontology enrichments were carried out same as what described for differential variants.

#### Linking variants to protein structure

Results of gene ontology enrichment analyses were applied for variants visualization. Some variants impact on a protein’s amino acid composition. Therefore, the protein’s three-dimensional structure was downloaded by CLC Genomic Workbench (8.5.1)^71^ from the protein data bank (www.rcsb.org) in order to show the variants location on the protein structure. Variants visualization was carried out for important differential candidate genes of our investigation’s results, or the ones that have their structures in the protein database structure.

#### Developing a new approach for genomic variants enrichment analysis

The identification of genomic regions that contained the highest density of differential variant between highland and lowland chickens, including both males and females, is the main purpose of genomic variants enrichment analysis. In this article, we proposed a plan to analyze the genomic variants enrichment based on the following steps. First, we estimated each chromosome’s standard frequencies of differential variants using the division of variants number by the chromosome length (Mb). Second, the chromosome that had the most standard frequency was selected, and the cumulative frequencies graph of variants was drawn along the chromosome by the R program (version 3.2.2)^79^. Third, chromosome region, which had the most cumulative frequency, was identified and ENSGALT (Ensembl transcript) IDs were also detected by UCSC and Table browser (www.genome.ucsc.edu/cgi-bin/hgTables) and finally, all detected ENSGALT IDs were converted into the ENSGAL gene ID by BioMart (www.ensembl.org/biomart).

#### Gene network analysis

According to all candidate genes identified in gene ontology enrichment analyses for differential variants, we carried out the gene network analysis. A total of 27 candidate genes was suggested for differential variants analysis among males and females (Tables 7 and 8). In order to build an informative gene network, chicken Ensembl gene identifications were converted into human orthologs^72^. Based on the following parameters, gene network analysis was carried out by Studio pathway web (Elsevier) for the identification of upstream neighbor networks for diseases: Min overlap = 2, and P value < 0.05^86^.

#### Validations

Variants discovery projects produce numerous variations. Thereby, validating the variants is highly required. Here, validations were carried out for two groups of detected variants. First, novel differential SNVs between highland and lowland chickens, and second, mtDNA variations in highland chickens. Thus, the sequencing of other five whole genomes of highland samples (Isfahan) was carried out, separately. The whole genome sequencing, trimming, and variant detection were performed according to the information provided in the material and method sections. A total of 3845 SNVs was reported as new variants for differential variants analyses and 32 variations were also detected in mtDNA for highland samples (see result part). Each SNV’s regions and chromosomes were evaluated in the new five samples by R program in order to validate the variants^79^. The percentage of validation was calculated based on those samples that had variants.

## Supplementary information

Supplementary information

Dataset 1

Dataset 2

Dataset 3

Dataset 4

Dataset 5

Dataset 6

Dataset 7

Dataset 8

Dataset 9

Dataset 10

Dataset 11

## Data Availability

Produced and analyzed datasets of this study are not publicly available due to the policy of financial sponsor. But datasets are available from the corresponding author on reasonable request. In addition, novel detected SNVs with an accession number of PRJEB24944 are available in EBI.

## References

[1] Tixier-Boichard M, Bed’hom B, Rognon X (2011). Chicken domestication: From archeology to genomics. C R Biol..

[2] Burt DW (2005). Chicken genome: current status and future opportunities. Genome Res..

[3] Meydan H, Jang CP, Yıldız MA, Weigend S (2016). Maternal origin of Turkish and Iranian native chickens inferred from mitochondrial DNA D-loop sequences. Asian Austral J Anim Sci..

[4] Shahbazi S, Mirhosseini SZ, Romanov MN (2007). Genetic diversity in five Iranian native chicken populations estimated by microsatellite markers. Biochem Genet..

[5] Barba M, Czosnek H, Hadidi A (2014). Historical perspective, development and applications of next-generation sequencing in plant virology. Viruses..

[6] Yan Y, Yi G, Sun C, Qu L, Yang N (2014). Genome-wide characterization of insertion and deletion variation in chicken using next generation sequencing. PloS One.

[7] Grocott M, Montgomery H, Vercueil A (2007). High-altitude physiology and pathophysiology: implications and relevance for intensive care medicine. Crit Care..

[8] De B (2013). Systems biology approach to study the high altitude adaptation in tibetans. Braz Arch Biol Techn..

[9] Jia C (2016). Gene Co-Expression Network Analysis Unraveling Transcriptional Regulation of High-Altitude Adaptation of Tibetan Pig. PloS One.

[10] Dong K (2014). Genomic scan reveals loci under altitude adaptation in Tibetan and Dahe pigs. PLoS One.

[11] Srivastava S (2012). Association of polymorphisms in angiotensin and aldosterone synthase genes of the renin–angiotensin–aldosterone system with high-altitude pulmonary edema. J. Renin Angiotensin Aldosterone Syst..

[12] Michiels C (2004). Physiological and pathological responses to hypoxia. Am J Pathol..

[13] Takiyama Yumi, Haneda Masakazu (2014). Hypoxia in Diabetic Kidneys. BioMed Research International.

[14] Stobdan T, Karar J, Pasha MQ (2008). High altitude adaptation: genetic perspectives. High Alt Med Biol..

[15] Zhang Q (2016). Genome resequencing identifies unique adaptations of Tibetan chickens to hypoxia and high-dose ultraviolet radiation in high-altitude environments. Genome Biol Evol..

[16] Zhao X (2016). High-altitude adaptation of Tibetan chicken from MT-COI and ATP-6 perspective. Mitochondrial DNA..

[17] Vasiev B, Balter A, Chaplain M, Glazier JA, Weijer CJ (2010). Modeling gastrulation in the chick embryo: formation of the primitive streak. PLoS One.

[18] Takeuchi T, Watanabe Y, Takano‐Shimizu T, Kondo S (2006). Roles of jumonji and jumonji family genes in chromatin regulation and development. Dev Dyn..

[19] Wilkanowska A, Mazurowski A, Mroczkowski S, Kokoszyński D (2014). Prolactin (PRL) and prolactin receptor (PRLR) genes and their role in poultry production traits. Folia biologica..

[20] Hsieh DJ-Y (2015). Prolactin protects cardiomyocytes against intermittent hypoxia-induced cell damage by the modulation of signaling pathways related to cardiac hypertrophy and proliferation. Int J Cardiol..

[21] Felgentreff K (2015). Functional analysis of naturally occurring DCLRE1C mutations and correlation with the clinical phenotype of ARTEMIS deficiency. J Allergy Clin Immunol..

[22] Ebrahimie E, Ebrahimi F, Ebrahimi M, Tomlinson S, Petrovski KR (2018). Hierarchical pattern recognition in milking parameters predicts mastitis prevalence. Comput Electron Agr..

[23] Sharifi S (2018). Integration of machine learning and meta-analysis identifies the transcriptomic bio-signature of mastitis disease in cattle. PloS One.

[24] Hartmut M (1999). Cytochrome c oxidase: catalytic cycle and mechanisms of proton pumping-A discussion. Biochem..

[25] Sun JS, Zhong H, Chen SY, Yao YG, Liu YP (2013). Association between MT-CO3 haplotypes and high-altitude adaptation in Tibetan chicken. Gene..

[26] Camus MF, Wolf JB, Morrow EH, Dowling DK (2015). Single Nucleotides in the mtDNA Sequence Modify Mitochondrial Molecular Function and Are Associated with Sex-Specific Effects on Fertility and Aging. Curr Biol..

[27] Mossman JA, Tross JG, Li N, Wu Z, Rand DM (2016). Mitochondrial-Nuclear Interactions Mediate Sex-Specific Transcriptional Profiles in Drosophila. Genetics..

[28] Lemarie A, Grimm S (2011). Mitochondrial respiratory chain complexes: apoptosis sensors mutated in cancer?. Oncogene..

[29] Zhou F, Yin Y, Su T, Yu L, Yu C-A (2012). Oxygen dependent electron transfer in the cytochrome bc1 complex. Biochimica et Biophysica Acta (BBA)-Bioenergetics..

[30] Liu L, Simon MC (2004). Regulation of Transcription and Translation by Hypoxia. Cancer Biol Ther..

[31] Koritzinsky M, Wouters BG (2007). Hypoxia and regulation of messenger RNA translation. Methods Enzymol..

[32] Staudacher JJ (2015). Hypoxia-induced gene expression results from selective mRNA partitioning to the endoplasmic reticulum. Nucleic Acids Res..

[33] Tamagnone L (1994). BMX, a novel nonreceptor tyrosine kinase gene of the BTK/ITK/TEC/TXK family located in chromosome Xp22. 2. Oncogene.

[34] Paavonen K (2004). Bmx tyrosine kinase transgene induces skin hyperplasia, inflammatory angiogenesis, and accelerated wound healing. Mol Biol Cell..

[35] McNamee EN, Johnson DK, Homann D, Clambey ET (2013). Hypoxia and hypoxia-inducible factors as regulators of T cell development, differentiation, and function. Immunol Res..

[36] Xia X (2009). Integrative analysis of HIF binding and transactivation reveals its role in maintaining histone methylation homeostasis. Proceedings of the National Academy of Sciences.

[37] Niu X (2012). The von Hippel–Lindau tumor suppressor protein regulates gene expression and tumor growth through histone demethylase JARID1C. Oncogene..

[38] Hancock RL, Dunne K, Walport LJ, Flashman E, Kawamura A (2015). Epigenetic regulation by histone demethylases in hypoxia. Epigenomics..

[39] Zhang H, Burggren W (2012). Hypoxic level and duration differentially affect embryonic organ system development of the chicken (Gallus gallus). Poult Sci..

[40] Mattiesen W-RC (2009). Increased neurogenesis after hypoxic-ischemic encephalopathy in humans is age related. Acta neuropathological..

[41] Patterson AJ, Zhang L (2010). Hypoxia and fetal heart development. Curr Mol Med..

[42] Joiner DM, Ke J, Zhong Z, Xu HE, Williams BO (2013). LRP5 and LRP6 in development and disease. Trends Endocrinol Metab..

[43] Svobodová AR (2012). DNA damage after acute exposure of mice skin to physiological doses of UVB and UVA light. Arch Dermatol Res..

[44] Yel M, Güven T, Türker H (2014). Effects of ultraviolet radiation on the stratum corneum of skin in mole rats. J. Radiat Res Appl..

[45] Zhang B (2015). A Comprehensive MicroRNA Expression Profile Related to Hypoxia Adaptation in the Tibetan Pig. PloS One.

[46] Blick C (2015). Identification of a hypoxia-regulated miRNA signature in bladder cancer and a role for miR-145 in hypoxia-dependent apoptosis. Brit J Cancer..

[47] Kulshreshtha R (2007). A microRNA signature of hypoxia. Mol Cell Biol..

[48] Shao P (2012). Drastic expression change of transposon-derived piRNA-like RNAs and microRNAs in early stages of chicken embryos implies a role in gastrulation. RNA Biol..

[49] Ghatpande SK, Billington CJ, Rivkees SA, Wendler CC (2008). Hypoxia induces cardiac malformations via A1 adenosine receptor activation in chicken embryos. Birth Defects Res A Clin Mol Terato..

[50] Lo KH, Hui MNY, Yu RMK, Wu RSS, Cheng SH (2011). Hypoxia impairs primordial germ cell migration in zebrafish (Danio rerio) embryos. PLoS One.

[51] Chakraborty C, Hsu CH, Wen ZH, Lin CS, Agoramoorthy G (2009). Zebrafish: a complete animal model for *in vivo* drug discovery and development. Curr Drug Metab..

[52] Burt DW (2007). Emergence of the chicken as a model organism: implications for agriculture and biology. Poult Sci..

[53] Dodgson, J. B. & Romanov, M. N. Use of chicken models for the analysis of human disease. *Curr Protoc Hum Genet*. 15.5. 1–15.5. 12 (2004). 10.1002/0471142905.hg1505s40 18428358

[54] Mathieu A-L (2015). PRKDC mutations associated with immunodeficiency, granuloma, and autoimmune regulator–dependent autoimmunity. J Allergy Clin Immunol.

[55] Blunt T (1996). Identification of a nonsense mutation in the carboxyl-terminal region of DNA-dependent protein kinase catalytic subunit in the scid mouse. Proceedings of the National Academy of Sciences.

[56] Xie W, Su Y-h, Feng Q, Qu L-k, Shou C-C (2015). Inhibitory effects of silencing PES1 gene expression on the malignant phenotypes of colon cancer cells. Tumor..

[57] Li J (2016). Repression of PES1 expression inhibits growth of gastric cancer. Tumor Biol..

[58] Brennan M, Lim B (2015). The Actual role of receptors as cancer markers, biochemical and clinical aspects: receptors in breast cancer. Adv Exp Med Biol..

[59] Bar-Shavit R (2016). G protein-coupled receptors in cancer. Int J Mol Sci..

[60] Li Y, Lu W, He X, Schwartz AL, Bu G (2004). LRP6 expression promotes cancer cell proliferation and tumorigenesis by altering β-catenin subcellular distribution. Oncogene..

[61] Liu C-C, Prior J, Piwnica-Worms D, Bu G (2010). LRP6 overexpression defines a class of breast cancer subtype and is a target for therapy. Proceedings of the National Academy of Sciences..

[62] Ma J, Lu W, Chen D, Xu B, Li Y (2017). Role of Wnt Co‐Receptor LRP6 in Triple Negative Breast Cancer Cell Migration and Invasion. J Cell Biochem..

[63] Tung EK-K, Wong BY-C, Yau T-O, Ng IO-L (2012). Upregulation of the Wnt co-receptor LRP6 promotes hepatocarcinogenesis and enhances cell invasion. PloS One.

[64] Calderwood Stuart K., Stevenson Mary Ann, Murshid Ayesha (2012). Heat Shock Proteins, Autoimmunity, and Cancer Treatment. Autoimmune Diseases.

[65] Lianos GD (2015). The role of heat shock proteins in cancer. Cancer letters..

[66] Yang Z (2015). Upregulation of heat shock proteins (HSPA12A, HSP90B1, HSPA4, HSPA5 and HSPA6) in tumour tissues is associated with poor outcomes from HBV-related early-stage hepatocellular carcinoma. Int J Med Sci..

[67] Wu J (2017). Heat shock proteins and cancer. Trends Pharmacol Sci..

[68] Rerole AL, Jego G, Garrido C (2011). Hsp70: anti-apoptotic and tumorigenic protein. Methods Mol Biol..

[69] Iranpur-Mobarakeh, V. Esmailizadeh, A, K. Rapid Extraction of High Quality DNA from Whole Blood Stored at -4 C for Long Period. *Protocol Online*, http://www.protocol-online.org (2010).

[70] Minitab 17 Statistical Software [Computer software]. State College, PA: Minitab, Inc., www.minitab.com (2010).

[71] CLC Genomics Workbench 8.5.1, https://www.qiagenbioinformatics.com/.

[72] Doan Ryan, Cohen Noah D, Sawyer Jason, Ghaffari Noushin, Johnson Charlie D, Dindot Scott V (2012). Whole-Genome sequencing and genetic variant analysis of a quarter Horse mare. BMC Genomics.

[73] Mortazavi A, Williams BA, McCue K, Schaeffer L, Wold B (2008). Mapping and quantifying mammalian transcriptomes by RNA-Seq. Nat Methods..

[74] Sims D, Sudbery I, Ilott NE, Heger A, Ponting CP (2014). Sequencing depth and coverage: key considerations in genomic analyses. Nat Rev Genet..

[75] Lander ES, Waterman MS (1988). Genomic mapping by fingerprinting random clones: a mathematical analysis. Genomics..

[76] Dias, M. *et al*. SNP detection using RNA-sequences of candidate genes associated with puberty in cattle. *Genet Mol Res*. **16**, 10.4238/gmr16019522 (2017). 10.4238/gmr16019522 28340271

[77] Romualdi C, Bortoluzzi S, d’Alessi F, Danieli GA (2003). IDEG6: a web tool for detection of differentially expressed genes in multiple tag sampling experiments. Physiol Genomics..

[78] Yeh FC, Yang RC, Boyle TB, Ye Z, Mao JX (1997). POPGENE, the user-friendly shareware for population genetic analysis. Molecular biology and biotechnology centre, University of Alberta, Canada.

[79] R Core Team R: A language and environment for statistical computing. R Foundation for Statistical Computing, Vienna, Austria. URL, http://www.R-project.org/ (2014).

[80] Nenadic, O. & Greenacre, M. Correspondence analysis in R, with two-and three-dimensional graphics: The ca package. *J Stat Softw*. **20**, 10.18637/jss.v020.i03 (2007).

[81] Abdi, H. &Valentin, D. Multiple correspondence analysis. *Encyclopedia of measurement and statistics*, 651–657 (2007).

[82] Comparative analysis of three bovine genomes, www.qiagenbioinformatics.com/support/tutorials (2017).

[83] Weir BS, Cockerham CC (1984). Estimating F-Statistics for the Analysis of Population Structure. Evolution..

[84] Ming-Shan W (2015). Genomic Analyses Reveal Potential Independent Adaptation to High Altitude in Tibetan Chickens. Mol Biol Evol..

[85] Nosrati Maryam, Asadollahpour Nanaei Hojjat, Amiri Ghanatsaman Zeinab, Esmailizadeh Ali (2018). Whole genome sequence analysis to detect signatures of positive selection for high fecundity in sheep. Reproduction in Domestic Animals.

[86] Pashaiasl M, Ebrahimi M, Ebrahimie E (2016). Identification of the key regulating genes of diminished ovarian reserve (DOR) by network and gene ontology analysis. Mol Biol Rep..

