# Supplementary material for: Genomic analysis reveals variant association with high altitude adaptation in native chickens
Source: Sci Rep. 2019 Jun 25;9:9224. doi: 10.1038/s41598-019-45661-7 (PMC6592930; doi:10.1038/s41598-019-45661-7)
Supplement: Supplementary file 1 — Supplementary information [file 41598_2019_45661_MOESM1_ESM.pdf]

Genomic analysis reveals variant association with high altitude adaptation in native chickens

Hamed Kharrati-Koopaee[1], Esmaeil Ebrahimie[1,2,3,4*], Mohammad Dadpasand[5], Ali Niazi[1], Ali Esmailizadeh[6,7 *]

1. Institute of Biotechnology, School of Agriculture, Shiraz University, Shiraz, Iran.
2. The University of Adelaide, School of Medicine, Adelaide, South Australia, Australia.
3. School of Information Technology and Mathematical Science, Division of Information Technology, Engineering and the Environment, University of South Australia, South Australia, Adelaide, Australia.
4. School of Biological Science, Faculty of Science and Engineering, Flinders University, South Australia, Adelaide, Australia.
5. Department of Animal science, School of Agriculture, Shiraz University, Shiraz, Iran.

6.State Key Laboratory of Genetic Resources and Evolution, and Yunnan Laboratory of Molecular Biology of Domestic Animals, Kunming Institute of Zoology, Chinese Academy of Sciences No. 32 Jiaochang Donglu, Kunming, Yunnan, 650223, P.R. China.

7. Department of Animal science, Faculty of Agriculture, Shahid Bahonar University of Kerman, Kerman, Iran.

[*]Corresponding authors: 1. Esmaeil Ebrahimie

2. Ali Esmailizadeh

Supplementary text

Results

Common variants

Totally in males and females, 53780 variants were shared between highland and lowland chickens and 16835 new variants were reported for the first time. The number of 516 variants were identified that can lead to change in amino acid sequences (Supplementary Table S5 online).

The results of gene ontology enrichment analysis in females and males indicated that there were GO terms that related to cell survival. For example, cell proliferation, cell growth (*ROS1)*, digestion (*PRSS3)*, chromosome organization (*BRCA2)*, RNA processing *(TDRD9)*, telomere capping (*POT1)* and cytokinesis *(CEP55)*.

In females, centrosome (*BRCA2*) had the most frequency as main organelle for cellular component analysis, it contributes in cell division. Also, cell proliferation (*ROS1*) was identified as the most importance biological pathway.

In males, the results of molecular function analyses showed that damaged DNA binding protein (*MSH3*) was shared mostly between highland and lowland chickens and it involves in DNA repair process. Midbody structure (*CEP55*) and cell proliferation (*ROS1*) had the most frequency in cellular component and biological process analysis. More results of gene ontology analysis are shown in supplementary Tables S6 and S7 online.

Discussion

Common variants analysis indicated that shared variants are involved in cell survival, protein modification and reproduction. Several candidate genes such as *ROS1*, *PRSS3*, *BRCA2*, *TDRD9*, *POT1*, *CEP55* were detected for cell proliferation, digestion, chromosome organization, RNA processing, telomere capping and cytokinesis, respectively. In human, *BRCA2* (breast cancer 2) is involved in chromosomal damage and DNA repair. The mutated *BRCA2* gene leads to unrepair DNA and finally breast cancer[1]. Furthermore, we showed that the regulation of ERK (extracellular signal–regulated kinases) was shared between highland and lowland samples. ERK is involved in mitosis, meiosis and post meiotic function in different cells.

The results of gene ontology indicated that protein structure modification was shared between highland and lowland chickens. For example, protein depalmitoylation is a dynamic post translational modification. In this biological process, 16-carbons fatty acidis added to cysteine of protein and finally it is removed by acyl protein thioesterases (APTs). This modification leads to modulation of protein sorting, targeting and signaling[2]. Additionally, here we suggested *LYPLAL1* gene as a candidate gene for this biological process. *LYPLAL1* gene (Lysophospholipase like 1) has depalmitoylating activity and able to hydrolyze only short chain substrates due to its shallow active site (Entrez gene: 127018). In addition, the results of molecular function analysis showed that palmitoyl-(protein) hydrolase activity function can be considered for protein modification as was described. *CEP55* gene was reported for establishment of protein localization in cells.

Reproduction is one of the most important factors for species survival. Therefore, it seems logical that there should be common variants for reproduction between highland and lowland chickens. Our study identified *TDRD9* gene for P granule. Vornina (2013)[3], reported that P granule are conserved cytoplasmic organelles that are present in *C.elegans* and germ cells. In embryogenesis process, P granules are segregated asymmetrically into those blastomeres and finally the germ line is produced. There is correlation between P granules distribution and germ line developments. Consequently, P granules might have been associated with germ line specification and differentiation[4]. Also, in humans, *TDRD9* (Tudor Domain Containing 9) has critical role in spermatogenesis and germ line integrity (UniProtKB: Q8NDG6).

Differential variants analysis showed that adaptive variants were classified to ion channels, PeBoW complex and histone binding.

Previous studies have noted that there is association between cell viability and hypoxia condition. Ions channels have critical role in regulating most of the biological process (e.g., T cell activation, apoptosis, glucose metabolism, pancreatic β cell insulin release and transport of nutrients)[5-6]. Therefore, cell function depends on ions and their performance directly. K^+ channels are blocked by hypoxia condition, thus, the K^+ efflux is reduced in intracellular. Finally, it causes increased Ca^{2+} ion [5]. An increase in Ca^{2+} leads to cell growth, proliferation and inhibition of cell apoptosis[7]. In this study, *ATP13A4* gene (ATPase type13A4) was found to be associated with hypoxia adaptation. Vallipuram et al. (2010)[8] illustrated that *ATP13A4* gene may be involved in calcium regulation. They showed that over-expression of *ATP13A4* in COS-7

cells lead to increase in the intracellular calcium level. These findings further support the idea of Wang et al. (2015)[9] who reported that the genes involved in Ca^{2+} signaling pathway can be considered as candidate genes for adaptation of high altitude chickens to hypoxia.

PeBoW complex is checkpoint response between nucleus and cell cycles in mature cells. It is located in nucleus and involved in ribosome biogenesis specially 60s ribosomal subunit and cell proliferation. In mammalian cells, it consists of three proteins Pes1 (pescadillo), Bop1 (block of proliferation) and WDR12 (WD-repeat protein)[10-11]. In human cells, PeBow complex is linked to another process such as mitosis. For example, inactivation of Pes1 and Bop1 leads to abnormal mitosis [12]. Furthermore, over expression of PeBoW complex proteins has been detected in cancers [13]. Pervious evidence suggests that the signal stress such as hypoxia and heat shock causes disruption of PeBoW complex function and finally cell cycle process [14]. Consequently, our results showed that PeBoW complex can be considered as cellular component for hypoxia condition. In fact, differential variant analysis between highland and lowland samples illustrated that PeBoW complex may be involved in adaptation to high altitude condition. Also, *PES1* gene was detected for this GO term. The *PES1* gene which encodes pescadillo ribosomal biogenesis factor 1 which is one member of the PeBoW complex. It is necessary for maturation of 28S, 5.8S and 60S ribosomal subunits.

Several studies indicated that hypoxia condition might cause epigenetic regulation in cells. For example, histone methylation was reported in hypoxia[15-17]. The results of gene ontology showed that histone binding might have associated with hypoxia condition. Additionally, we suggested that *ATAD2* gene can be considered for histone binding process. *ATAD2* gene belongs to the ATPase family and is involved in chromatin binding and histone binding. This finding is in agreement with those of Zhang et al (2016)[18] who showed that there was association between histone binding and high altitude conditions.

In this study**,** sex chromosomes were also analyzed. Several GO terms were reported for common and differential variants. Differential variants analysis showed that there were two GO terms as molecular function in females on chromosome Z (Table 8). They included alanine-glyoxylate transaminase activity and pyridoxal phosphate binding. In addition, *AGXT2* gene was reported as candidate gene for these molecular functions. In fact, *AGXT2* encodes alanine-glyoxylate transaminase enzyme and it catalyzes the following biochemical reactions[19].

L-alanine + glyoxylate ⇌ pyruvate + glycine

Pyridoxal phosphate (vitamin B6) is coenzyme in this reaction. Under anaerobic condition, oxidative phosphorylation is inhibited, while glycolysis pathway is stimulated resulting in the production of pyruvate and lactate[20]. Also, Tsai et al. (2013)[21] showed that the amount of alanine is decreased in hypoxic cell compared with normoxic cells. In the other words, the decrease of alanine might be due to the conversion to pyruvate and finally ATP production.

In males, common variants analysis indicated several GO terms were associated with Y-form DNA binding, loop DNA binding and damaged DNA binding (Table S7). All of them are involved in DNA repair system. For example, DNA damage-binding is a complex protein that is responsible for DNA repair of UV-damaged DNA. This complex protein has a role in nucleotide excision repair system[22]. It seems reasonable, because previous studies demonstrated that UV radiation causes DNA damage in high-altitude condition[15]. Furthermore, *MSH3* gene was reported as candidate gene for this molecular function analysis. *MSH3* gene encodes DNA mismatch repair protein. In the other words, it forms a heterodimer with *MSH2* to form MutS beta and finally MutS beta initiates mismatch repair by binding to a mismatch base[23].

Supplementary references

1. Friedenson, B. The BRCA1/2 pathway prevents hematologic cancers in addition to breast and ovarian cancers. *BMC Cancer.* **7,** 152-163 (2007).

2. Lin, D. T. & Conibear, E. Enzymatic protein depalmitoylation by acyl protein thioesterases. *Biochem Soc Trans.* **43,** 193-198 (2015).

3. Voronina, E. The diverse functions of germline P-granules in *Caenorhabditis elegans*. *Mol Reprod Dev*. **80,** 624-631(2013).

4. Pitt, J. N., Schisa J. A. & Priess, J. R. P granules in the germ cells of *Caenorhabditis elegans* adults are associated with clusters of nuclear pores and contain RNA. *Dev Biol.* **219,** 315-333 (2000).

5. Shimoda, L. A. & Polak, J. Hypoxia. 4. Hypoxia and ion channel function. *Am J. Physiol Cell Physiol.* **300,** 951–967 (2010).

6. Prevarskaya, N., Skryma, R., Bidaux, G., Flourakis, M., Shuba, Y. Ion channels in death and differentiation of prostate cancer cells. *Cell Death Differ*. **14,** 1295–1304 (2007).

7. Ning, L., Wenju, L., Jian, W. Ca^{2+} and ion channels in hypoxia-mediated pulmonary hypertension. *Int J. Clin Exp Pathol*. **8,**1081-1092 (2015).

8. Vallipuram, J., Grenville, J. & Crawford, D. A. The E646D-ATP13A4 mutation associated with autism reveals a defect in calcium regulation. *Cell Mol Neurobiol*. **30,** 233-46 (2010).

9. Wang, M.S. Genomic analyses reveal potential independent adaptation to high altitude in Tibetan chickens. *Mol Biol Evol*. **32,** 1880–1889 (2015).

10. Rohrmoser, M. et al. Interdependence of Pes1, Bop1, and WDR12 controls nucleolar localization and assembly of the PeBoW complex required for maturation of the 60S ribosomal subunit. *Mol Cell Biol*. **27,** 3682-3694 (2007).

11. Holzel, M. et al. Mammalian WDR12 is a novel member of the Pes1-Bop1 complex and is required for ribosome biogenesis and cell proliferation. *J. Cell Biol.* **170,** 367–378 (2005).

12. Killian, A. et al. Inactivation of the RRB1-Pescadillo pathway involved in ribosome biogenesis induces chromosomal instability. *Oncogene*. **23,** 8597-8602 (2004).

13. Killian, A. et al. Contribution of the BOP1 gene, located on 8q24, to colorectal tumorigenesis. *Gene Chromosome Canc*. **45,** 874-881(2006).

14. Pfisterer, I. The role of the PeBoW-complex in ribosome biogenesis and proliferation of mouse embryonic stem cells. Dissertation, LMU München: Faculty of Biology. 15-16 (2007).

15. Xia, X. et al. Integrative analysis of HIF binding and transactivation reveals its role in maintaining histone methylation homeostasis. *Proc Natl Acad Sci*. **106,** 4260–4265 (2009).

16. Niu, X. et al. The von Hippel-Lindau tumor suppressor protein regulates gene expression and tumor growth through histone demethylase JARID1C. *Oncogene*. **31,** 776–786 (2012).

17. Hancock, R. L., Dunne, K., Walport, L. J., Flashman, E., Kawamura, A. Epigenetic regulation by histone demethylases in hypoxia. *Epigenomics*. **7,** 791-811 (2015).

18. Zhang, Q. et al. Genome resequencing identifies unique adaptations of Tibetan chickens to hypoxia and high-dose ultraviolet radiation in high-altitude environments. *Genome Biol Evol*. **8,** 765-776 (2016).

19. Nishijima, S., Sugaya, K., Morozumi, M., Hatano, T., Ogawa, Y. Hepatic alanine-glyoxylate aminotransferase activity and oxalate metabolism in vitamin B6 deficient rats. *J Urol*. **169,** 683-6(2003).

20. Southam, A. D. et al. Metabolic changes in flatfish hepatic tumours revealed by NMR-based metabolomics and metabolic correlation networks. *J. Proteome Res.* **7,** 5277–5285 (2008).

21. Tsai, I. L. et al. Metabolomic dynamic analysis of hypoxia in MDA-MB-231 and the comparison with inferred metabolites from transcriptomics data. *Cancers*. **5,** 491-510 (2013).

22. Yeh, J. I. et al. Damaged DNA induced UV-damaged DNA-binding protein (UV-DDB) dimerization and its roles in chromatinized DNA repair. *Proc Natl Acad Sci.* **109,** 2737-2746 (2012).

23. Gao, J. X. et al. MSH3 Mismatch Repair Protein Regulates Sensitivity to Cytotoxic Drugs and a Histone Deacetylase Inhibitor in Human Colon Carcinoma Cells. *PLoS ONE*. **8,** e65369. doi: 10.1371/journal.pone.0065369 (2013).

Supplementary Tables

Table S1: The parameter optimization for variants discovery

Parameters and variant number	Step 1	Step 2	Step 3
Required variant probability%	90	95	95
Ignore position with coverage above	100000	50000	50000
Minimum frequency	20	25	30
Neighborhood radius	5	10	15
Minimum central quality	20	25	30
Minimum neighborhood quality	15	20	25
The number of variation	2610510	2529822	2202492

The main goal of parameter optimizing was to decrease in false variants. In each step the threshold of parameters was increased and it leads to decrease in number of variation. Finally, parameters in step 3 were selected for variant detections.

Table S2: Percentage of discovered variants in highland and lowland chickens

Samples	Total variants	SNV	MNV	Insertion	Deletion	Replacement
Lowland	2355768	86.92	4.00	4.68	3.98	0.40
Lowland	2413224	86.28	3.89	5.02	4.37	0.42
Lowland	2147390	88.81	3.42	4.02	3.46	0.26
Lowland	1350667	88.83	3.62	3.91	3.37	0.25
Lowland	1965107	86.21	4.03	4.98	4.32	0.43
Highland	1947999	86.47	3.98	4.85	4.26	0.42
Highland	2475243	86.44	3.99	4.88	4.28	0.42
Highland	2207933	88.22	4.18	3.87	3.35	0.36
Highland	2075481	88.40	4.33	3.74	3.17	0.35
Highland	2228309	87.18	4.22	4.47	3.75	0.39

SNV: single nucleotide variation
MNV: multi nucleotide variation

Table S3: The results of comparing variants between highland and lowland samples for the identification of differential variants

Sex	Total variants	SNV	MNV	Insertion	Deletion	Replacement
Male	333262	280411	11049	16557	22755	2490
Male	419734	352375	13446	21422	29175	3316
Male	405642	349559	15680	15233	22726	2444
Female	248202	213114	10032	9704	13739	1613
Female	262688	222119	9511	12385	16643	2030

Variants of highland chickens were compared with reads of lowland chickens as a control tool in order to remove the common variation between lowland and highland samples. Then, a comparison was carried out within the male and female birds based on the frequency threshold optimization information (TableS4). Finally, differential variants are kept for further analysis including filtrations and gene ontology enrichment analysis.

Table S4: The summary of frequency threshold optimization for the detection of differential variants between highland and lowland chickens.

Frequency threshold%	0	25	50	75	100
The number of variations for males	1055762	1055762	**97610**	5927	5927
The number of variations for females	493996	493996	493996	17024	**17024**

Five frequency thresholds were considered in order to be utilized in the frequency threshold optimization (0, 25, 50, 75 and 100). The percentage of samples that have variants is considered as the threshold frequency. Thus, frequency thresholds of 50% and 100% were determined in order to make comparisons within males and females groups. Consequently, a total of 97610 and 17024 variants were detected as differential variants between highland and lowland samples.

Table S5: The results of common variant detection between highland and lowland chickens

Sex	Total variants	New variants	Coding variants	Amino acid changes
Male	35090	9810	504	270
Female	18690	7025	937	246
Total	53780	16835	1441	516

In current study, common variants were collected between highland and lowland chickens and classification of them were performed based on available annotations.
Novel variants: variants were reported for the first time.
Coding variants: variants were located in coding regions.
Amino acid changes: variations can change protein sequence.

Table S6: Results of gene ontology enrichment analysis for common variants in females

GO	Go term	Description	Gene	Chr	Type
Molecular function	0003697	Gamma-tubulin binding(7%)	*BRCA2*	1	SNV
	0003697	Single-stranded DNA binding(23%)	*BRCA2*	1	SNV
	0004713	Protein tyrosine kinase activity(9%)	*ROS1*	3	SNV
	0005487	Nucleocytoplasmic transporter activity(9%)	*TPR*	8	SNV
	0004252	Serine-type endopeptidase activity(52%)	*PRSS3*	1	SNV
Cellular component	0005643	Nuclear pore(28%)	*TPR*	8	SNV
	0005634	Centrosome(72%)	*BRCA2*	1	SNV
Biological Process	0051276	Chromosome organization(1%)	*BRCA2*	1	SNV
	0006913	Nucleocytoplasmic transport(4%)	*TPR*	8	SNV
	0016049	Cell growth(6%)	*ROS1*	3	SNV
	0070372	Regulation of ERK1 and ERK2 cascade(6%)	*ROS1*	3	SNV
	0007586	Digestion(7%)	*PRSS3*	1	SNV
	0051298	Centrosome duplication(9%)	*BRCA2*	1	SNV
	0018108<br>0008283	Peptidyl-tyrosine phosphorylation(18%)<br>Cell proliferation(25%)	*ROS1*	3	SNV
	0000724	Double-strand break repair via homologous recombination(24%)	*BRCA2*	1	SNV

Table S7: Results of gene ontology enrichment analysis for common variants in males

GO	Go term	Description	Gene	Chr	Type
Molecular function	0000403	Y-form DNA binding(3%)	*MSH3(Lys 417 Glu)*	Z	SNV
	0000404	Loop DNA binding(3%)			
	0003684	Damaged DNA binding(23%)			
	0008474	Palmitoyl-(protein) hydrolase activity(5%)	*LYPLAL1(Asn190Ser)*	3	SNV
	0052689	Carboxylic ester hydrolase activity(16%)			
	0005086	ARF guanyl-nucleotide exchange factor activity(10%)	*AMPH*	2	SNV
	0008519	Ammonium transmembrane transporter activity(8%)	*RHD*	23	Deletion
	0003697	Single-stranded DNA binding (23%)	*PMS1*	7	SNV
	0004713	Protein tyrosine kinase activity(9%)	*ROS1*	3	SNV
Cellular component	0032302	MutSbeta complex(4%)	*MSH3(Lys 417 Glu)*	Z	SNV
	0005712	Chiasma(6%)	*PMS1*	7	SNV
	0032389	MutLalpha complex(6%)			
	0000795	Synaptonemal complex(9%)			
	0008021	Synaptic vesicle(12%)	*AMPH*	2	SNV
	0030496	Midbody(14%)	*CEP55*	6	SNV
	0000783	Nuclear telomere cap complex(6%)	*POT1*	1	SNV
	0000781	Chromosome, telomeric region(15%)			
	0043186	P granule(13%)	*TDRD9*	5	SNV
	0000176	Nuclear exosome (RNase complex)(15%)	*MPHOSPH6*	11	SNV
Biological Process	0006298	Mismatch repair (12%)	*PMS1*	7	SNV
	0000281	Cytokinesis after mitosis(1%)	*CEP55*	6	SNV
	0045184	Establishment of protein localization(1%)			
	0000460	Maturation of 5.8S rRNA(3%)	*MPHOSPH6*	1	SNV
	0016233	Telomere capping(1%)	*POT1*	1	SNV
	0043570	Maintenance of DNA repeat elements(4%)	*MSH3(Lys 417 Glu)*	Z	SNV
	0002084	Protein depalmitoylation(5%)	*LYPLAL1(Asn190 Ser)*	3	SNV
	0006939	Smooth muscle contraction(5%)	*MYLK*	7	SNV
	0016049	Cell growth(5%)	*ROS1*	3	SNV
	0008283	Cell proliferation(20%)			
	0070372	Regulation of ERK1 and ERK2 (5%)			
	0015695	Organic cation transport(8%)	*RHD*	23	Deletion
	0006995	Cellular response to nitrogen starvation(15%)			
	0006396	RNA processing(15%)	*TDRD9*	5	SNV

Table S8: the results of gene network analysis for carcinogenesis in hypoxia condition

Disease	Total Genes	Overlap	Gene name	P-value
Carcinogenesis	5401	7	*PRKDC,ATAD2,PES1,DCLRE1A,BMX,LRP6,HSPA14*	0.001

Table S9: The information of submitted variants to EBI database

Description	Chromosome	Counts	type
Novel differential variants between highland and lowland chickens - females	Z	112	SNV
Novel differential variants between highland and lowland chickens - males		276	
Novel differential variants between highland and lowland chickens - females	Somatic	591	
Novel differential variants between highland and lowland chickens -males		2851	
Novel mt-DNA variants, highland chickens-female	Mt-DNA	8	
Novel mt-DNA variants, highland chickens-male		21	
Novel mt-DNA variants, lowland chickens-female		10	
Novel mt-DNA variants, lowland chickens-male		8	

In this study, differential variants between highland and lowland chicken, and mt-DNA variants were filtered based on known variant annotation. Consequently, many variants are discovered as novel variants for the first time. Finally, a total of 3877 SNVs variants are collected and are also submitted to EBI as novel variants with an accession number of PRJEB24944, for the first time

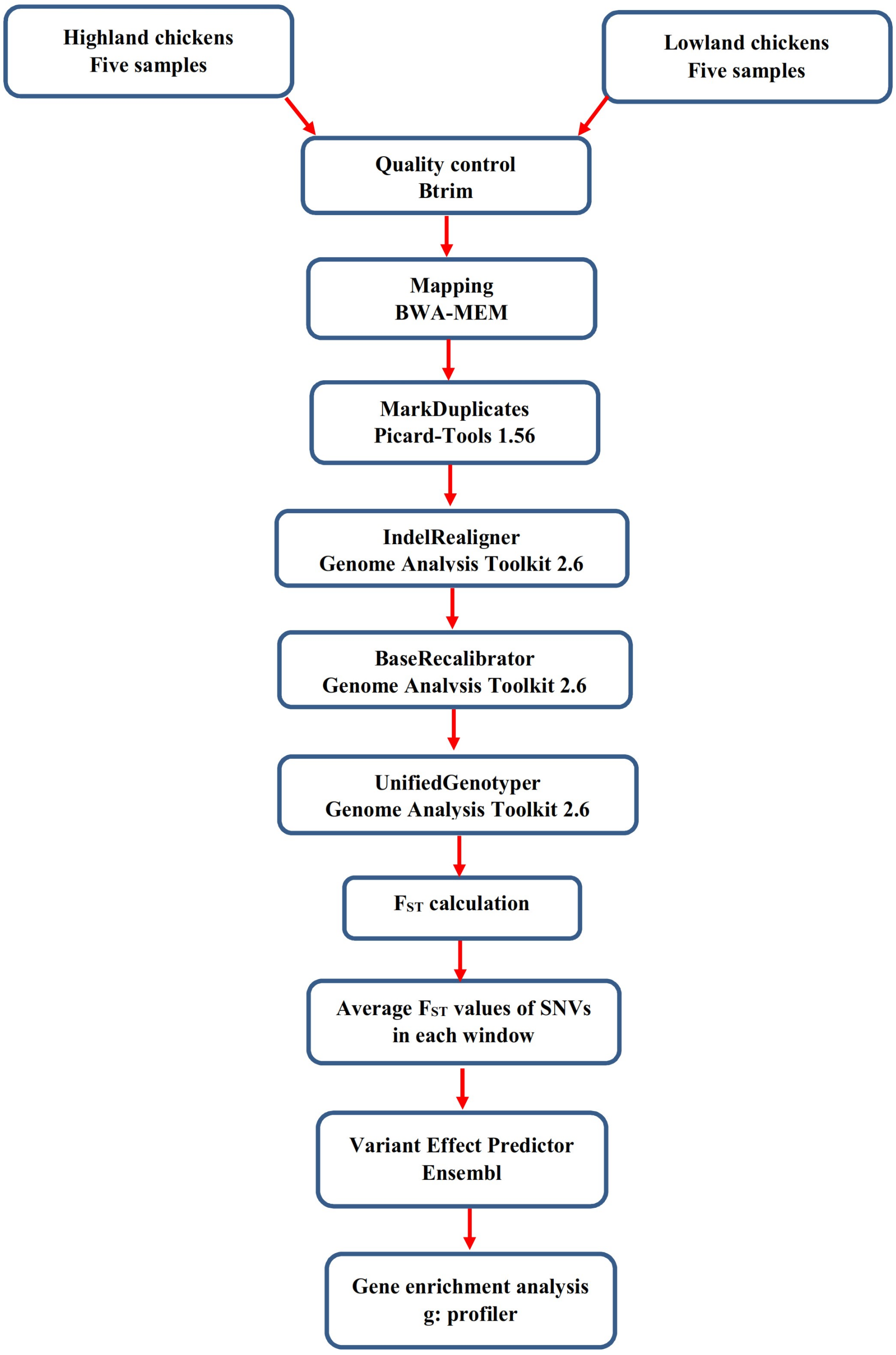

Figure S1: The Summary of signature selection analysis of native chicken ecotypes based on F_{ST} calculation.
